# miR‐181a regulates p62/SQSTM1, parkin, and protein DJ‐1 promoting mitochondrial dynamics in skeletal muscle aging

**DOI:** 10.1111/acel.13140

**Published:** 2020-04-15

**Authors:** Katarzyna Goljanek‐Whysall, Ana Soriano‐Arroquia, Rachel McCormick, Caroline Chinda, Brian McDonagh

**Affiliations:** ^1^ Discipline of Physiology School of Medicine National University of Ireland Galway Ireland; ^2^ Department of Musculoskeletal Biology Institute of Ageing and Chronic Disease University of Liverpool Liverpool UK

**Keywords:** aging, miR‐181a, mitophagy, p62, parkin, protein DJ‐1, skeletal muscle

## Abstract

One of the key mechanisms underlying skeletal muscle functional deterioration during aging is disrupted mitochondrial dynamics. Regulation of mitochondrial dynamics is essential to maintain a healthy mitochondrial population and prevent the accumulation of damaged mitochondria; however, the regulatory mechanisms are poorly understood. We demonstrated loss of mitochondrial content and disrupted mitochondrial dynamics in muscle during aging concomitant with dysregulation of miR‐181a target interactions. Using functional approaches and mito‐QC assay, we have established that miR‐181a is an endogenous regulator of mitochondrial dynamics through concerted regulation of Park2, p62/SQSTM1, and DJ‐1 in vitro. Downregulation of miR‐181a with age was associated with an accumulation of autophagy‐related proteins and abnormal mitochondria. Restoring miR‐181a levels in old mice prevented accumulation of p62, DJ‐1, and PARK2, and improved mitochondrial quality and muscle function. These results provide physiological evidence for the potential of microRNA‐based interventions for age‐related muscle atrophy and of wider significance for diseases with disrupted mitochondrial dynamics.

## Graphical abstract

We identified a global regulator of mitochondrial dynamics during skeletal muscle aging, miR‐181a. Downregulation of miR‐181a with age in skeletal muscle was associated with disrupted mitochondrial dynamics. miR‐181a targets regulators of autophagic machinery in C2C12 and promotes mitochondrial turnover. Restoration of miR‐181a in old muscle restores myofiber size and force.

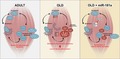

## 1 INTRODUCTION

Disrupted mitochondrial dynamics is one of the hallmarks of aging (Lopez‐Otin, Blasco, Partridge, Serrano, & Kroemer, 2013). Altered mitochondrial morphology and content in skeletal muscle of humans and rodents are one of the pathways consistently associated with age‐related loss of muscle mass and function (Bratic & Larsson, 2013; Short et al., 2005), with up to a 30% loss of mitochondrial content reported in fast twitch muscles and an accumulation of dysfunctional mitochondria (Chabi et al., 2008). Moreover, accumulation of damaged or dysfunctional mitochondria increases the oxidation of contractile proteins in muscle, associated with disrupted balance between anabolic and catabolic processes and ultimately sarcopenia (Carnio et al., 2014; O'Leary, Vainshtein, Iqbal, Ostojic, & Hood, 2013).

Mitochondria are dynamic organelles that exist in a highly interconnected network and are continually undergoing fusion and fission (for review, see Archer, 2013). These processes are tightly regulated and necessary to maintain a healthy mitochondrial population by removing and preventing the accumulation of damaged mitochondria. The regulation of mitochondrial remodeling and associated bioenergetic changes, particularly in skeletal muscle, is key for the correct adaptation and response to exercise that results in an increased mitochondrial content with improved fatty acid oxidation and glucose homeostasis (Mansueto et al., 2017). Moreover, the adaptive cellular response of skeletal muscle to exercise requires the autophagic degradation of cellular components, allowing the muscle fiber to rebuild and respond to repetitive bouts of exercise (Vainshtein, Grumati, Sandri, & Bonaldo, 2014). Interruption of mitochondrial dynamics can result in mitochondrial swelling, loss of cristae, destruction of the inner membrane, and impaired respiration (Bratic & Larsson, 2013). A decrease in the expression of regulators of mitochondrial fusion (Mfn1, Mfn2, and Opa1) and fission (Drp1) has been reported in sedentary old individuals, but in senior sportsmen that have maintained an active lifestyle their expression levels are comparable to younger individuals (Tezze et al., 2017). The integration of mitochondrial biogenesis and selective degradation *via* mitophagy is essential for the preservation of healthy muscle, and disruption of this balance can result in alterations in muscle bioenergetics and loss of muscle mass and function (Hood, Memme, Oliveira, & Triolo, 2019). Mitophagy is regulated at numerous levels, and a number of distinct mitophagic pathways have been elucidated such as ubiquitin‐mediated mitophagy including the Pink/Parkin pathway and ubiquitin independent pathways *via* mitophagy receptors on the outer mitochondrial membrane (e.g., BNIP3), however, the exact regulatory mechanisms remain to be fully understood (for reviews, see Montava‐Garriga & Ganley, 2019; Palikaras, Lionaki, & Tavernarakis, 2018).

microRNAs (miRs) are small 19–25 nt long noncoding RNAs that regulate gene expression post‐transcriptionally through binding to complementary target sites within mRNAs, usually 3′UTRs, leading to mRNA degradation and/or inhibition of mRNA translation (Bethune, Artus‐Revel, & Filipowicz, 2012). miRs target multiple genes and are considered a robust mechanism of controlling cellular and tissue homeostasis. The role of miRs in the regulation of key cellular mechanisms has become increasingly recognized, including skeletal muscle homeostasis, development, regeneration, and atrophy (Cheung et al., 2012; Goljanek‐Whysall et al., 2011; Soares et al., 2014). The expression of a number of specific miRs changes in skeletal muscle during exercise and aging (Kim et al., 2014; Nielsen et al., 2010). Although limited, functional studies have demonstrated that miRs play a key role in regulating the expression of genes and pathways altered during exercise and/or aging, contributing to alterations in skeletal muscle mass (Li, Chan, Yu, & Zhou, 2017; Silva, Bye, el Azzouzi, & Wisløff, 2017; Soares et al., 2014).

In this study, we have demonstrated that age‐related disruption of mitochondrial dynamics in skeletal muscle can be improved by restoring the expression of miR‐181a‐5p (miR‐181a). Quantitative proteomic data revealed a reduced mitochondrial protein content with age, concomitant with the upregulation of mitophagy‐associated proteins. Ultrastructural analysis of mitochondria revealed abnormal, large mitochondria in muscle during aging despite increased expression of autophagy‐, and in particular mitophagy‐associated proteins. Parallel analyses of upstream regulators of mitochondrial dynamics identified miR‐181a as targeting key autophagy‐ and mitochondrial dynamics‐associated genes*. *In vitro experiments confirmed miR‐181a targets p62 and Park2, and demonstrated that miR‐181a regulates mitophagic flux. Restoration of miR‐181a content in muscle of old mice in vivo prevented accumulation of p62, PARK2, and DJ‐1 and preserved mitochondrial content, ultimately resulting in increased myofiber size and muscle force. Together, our data indicate that miR‐181a is a potent regulator of muscle mitochondrial dynamics in vitro and in vivo*,* providing potential therapeutic avenues for age‐related muscle atrophy.

## 2 RESULTS

### 2.1 Quantitative proteomics reveals decrease in mitochondrial content with age

**Figure 1:**
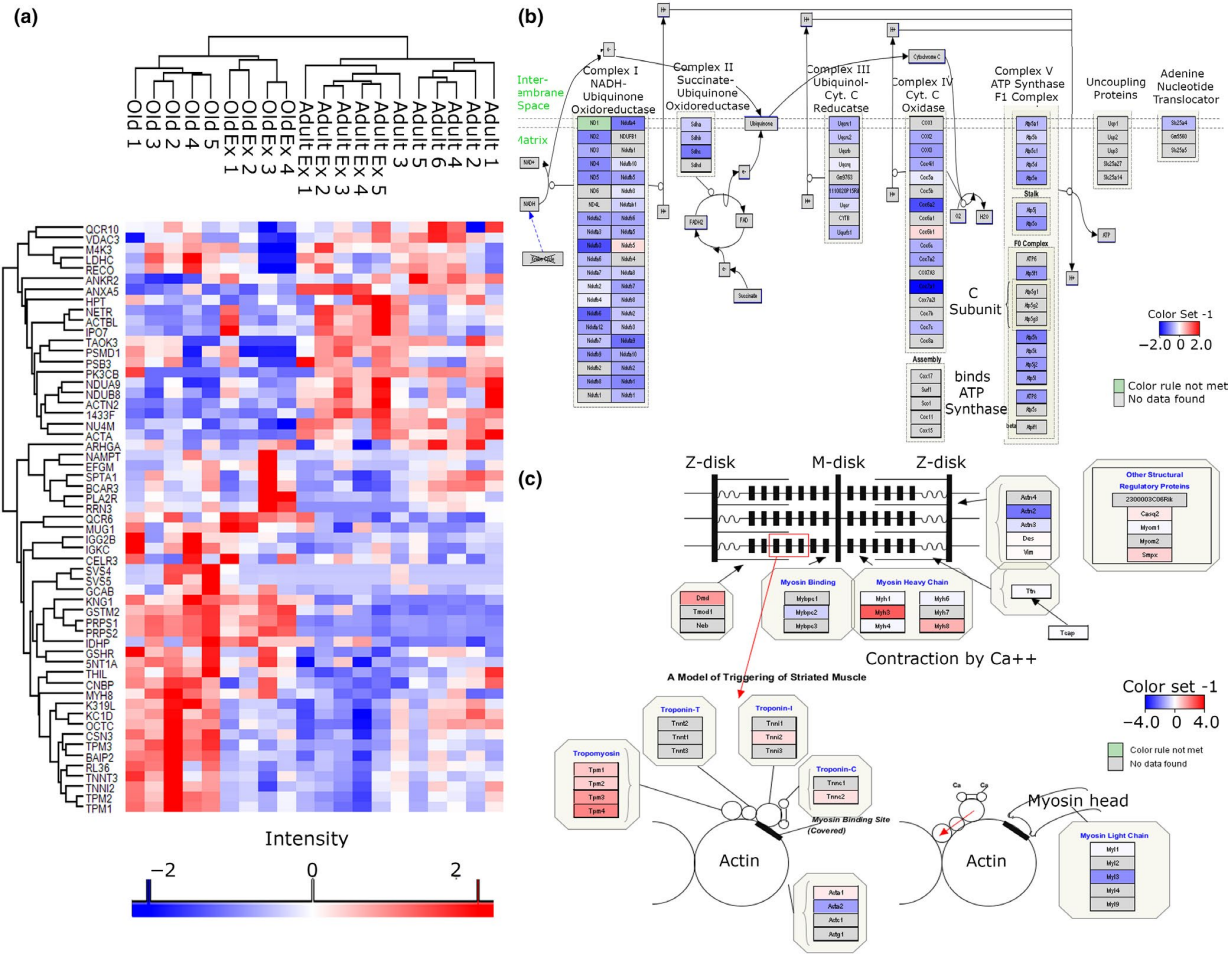
Label‐free proteomics of TA muscle from adult and old mice. (a) Heatmap of significantly changed proteins (fold change >2, −10log*P* > 20 equivalent to *p*‐value < .01) between adult (adult: 6 months old, *n* = 6), adult exercised (adult Ex, *n* = 5), old (old: 24 months old, *n* = 5), and old exercised (old Ex, *n* = 4). (b, c) Pathway analysis of quantitative proteomic data reveals a downregulation of proteins involved in mitochondrial electron transport chain and an upregulation of proteins involved in muscle contraction during aging

To characterize changes in the intracellular muscle environment during aging and associated adaptive response of muscle to contractions, global label‐free analysis was used to quantify the overall changes in the proteome of skeletal muscle from quiescent *tibialis anterior* (TA) or TA subjected to 15 min of isometric contractions (mimicking acute exercise) from adult and old mice. Significantly changed proteins (fold change >2 and −10log*P* > 20) between quiescent or contracted muscle of adult or old mice demonstrate clear differences in the proteomic content of TA muscle (Figure 1a). Despite detected changes in the abundance of some proteins between contracted muscle of adult and old mice, the major significant proteomic changes detected were as a result of aging. The most significantly changed pathways in muscle during aging were downregulation of mitochondrial proteins and upregulation of contractile apparatus proteins with age (Figure 1b,c). This is consistent with previously reported data showing mitochondrial content decrease in muscle during aging (Chabi et al., 2008; Hepple, 2014; Smith, Soriano‐Arroquia, Goljanek‐Whysall, Jackson, & McDonagh, 2018).

### 2.2 Autophagy regulatory and effector proteins are more abundant in skeletal muscle from old mice

**Figure 2:**
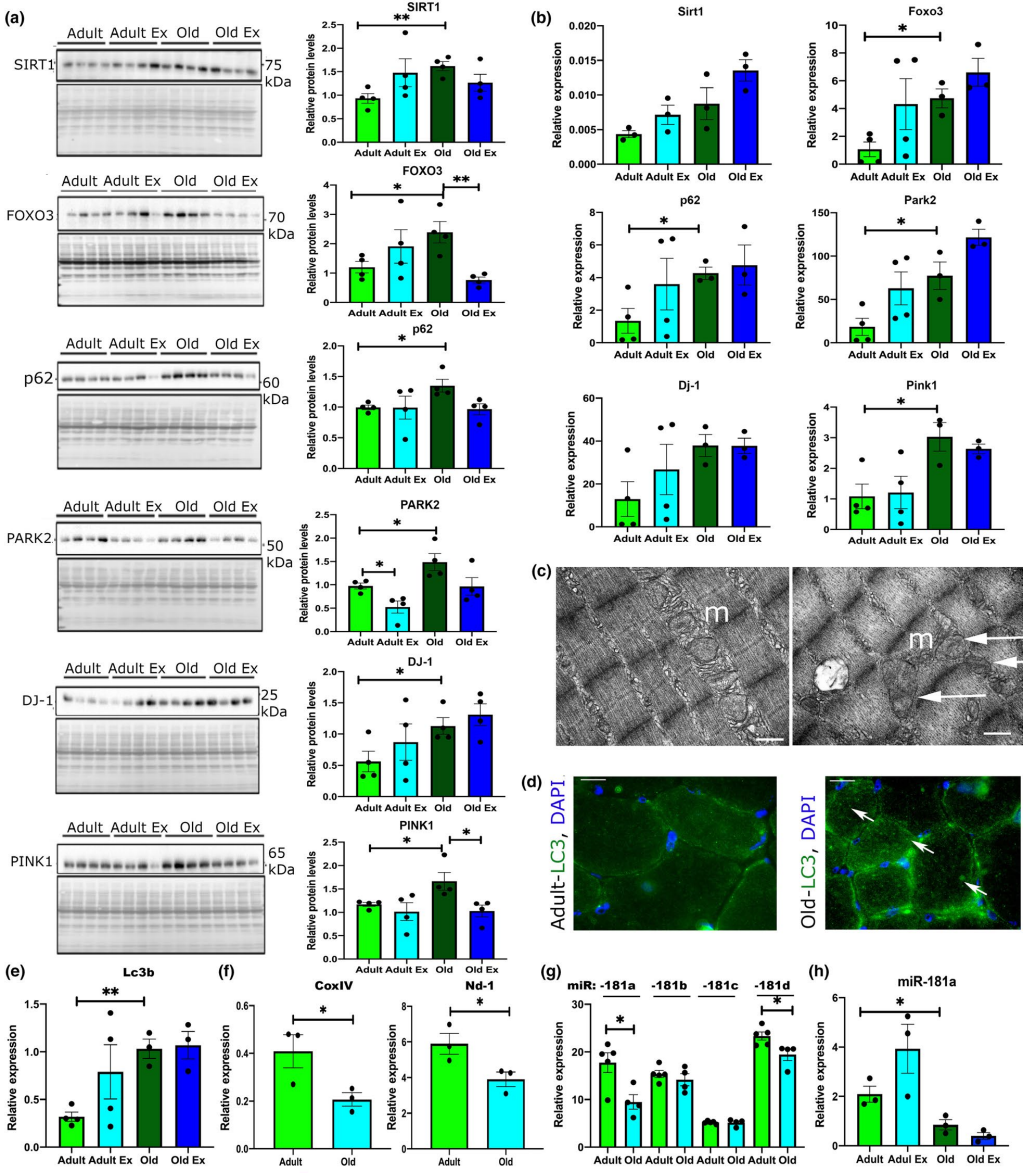
Autophagy and mitochondrial regulators have altered expression in skeletal muscle during aging. (a) Western blot analyses showing changes in the protein levels of autophagy‐associated proteins in the TA muscle of adult and old male mice. Levels normalized to Ponceau S (protein) are shown in the quantification graphs (arbitrary units). (b) Expression levels of mRNA of autophagy‐associated gene expression. (c) Representative EM images of mitochondria from TA muscles of adult and old mice; m—mitochondria; arrows indicate swollen or mitochondria with cristae in disarray. Scale bar: 0.5 µm. (d, e) Immunostaining (d) and qPCR (e) for LC3 on TA muscle of adult and old mice. Arrows indicate myofibers positive for LC3. Scale bars indicate 100 µm. Arrows indicate LC3‐positive fibers. (f) qPCR analyses of the expression of mitochondrial content markers encoded by the mitochondrial genome. (g) qPCR analyses of miR‐181a, miR‐181b, mR‐181c, and miR‐181d expression in the TA of adult and old mice. (h) qPCR analysis of miR‐181a expression in quiescent TA muscle and following contractions protocol of adult and old mice. Adult—6 months old; old—24 months old male C57BL6/J mice. Ex—TA following isometric contraction protocol. qPCR: Expression relative to β2‐microglobulin (genes) or Rnu6 (miR) is shown. Representative images are shown. *n* = 3–6. Error bars show *SEM*. *p < .05 Student's *t* test

To further investigate the mechanisms leading to decreased mitochondrial content during aging, we analyzed the expression of known regulators of autophagy and mitochondrial dynamics by Western blotting and qPCR. General regulators of the autophagic machinery: Sirtuin 1 (SIRT‐1) and Forkhead box protein O3 (FOXO3), as well as effector mitophagy proteins, such as PTEN‐induced putative kinase 1 (PINK1), Parkin (PARK2), Protein DJ‐1 (PARK7), and the autophagic adaptor protein p62 (Sequestosome 1, Sqstm1), were upregulated in muscle during aging (Figure 2a,b). We also observed downregulation of the expression of Pgc1α with age (Figure S1). Despite upregulation of autophagy‐associated proteins, swollen and abnormal mitochondria were detected by EM in muscle of old mice, suggesting defective mitochondrial dynamics (Figure 2c). This suggests impaired or dysfunctional autophagic response and mitochondrial biogenesis in skeletal muscle from old compared to adult mice, and an increase in p62 levels can be associated with an inhibition of autophagy as it is degraded in cells with normal autophagic flux (Figure 2b). Analysis of an autophagy marker LC3 revealed more pronounced LC3 punctae in muscle from old mice (Figure 2d). The expression of Lc3b was upregulated, and the levels of CoxIV and Nd‐1, indicators of mitochondrial content, were downregulated in the muscle of old mice, further suggesting dysfunctional autophagic response in aging muscle and in the adaptation to exercise (Figure 2e,f). Together, our data demonstrate an accumulation of key regulators of the mitophagic machinery in muscle during aging and defective mitochondrial dynamics resulting in accumulation of abnormal mitochondria and loss of mitochondrial content.

### 2.3 miR‐181a as putative regulator of mitochondrial dynamics

**Figure 3:**
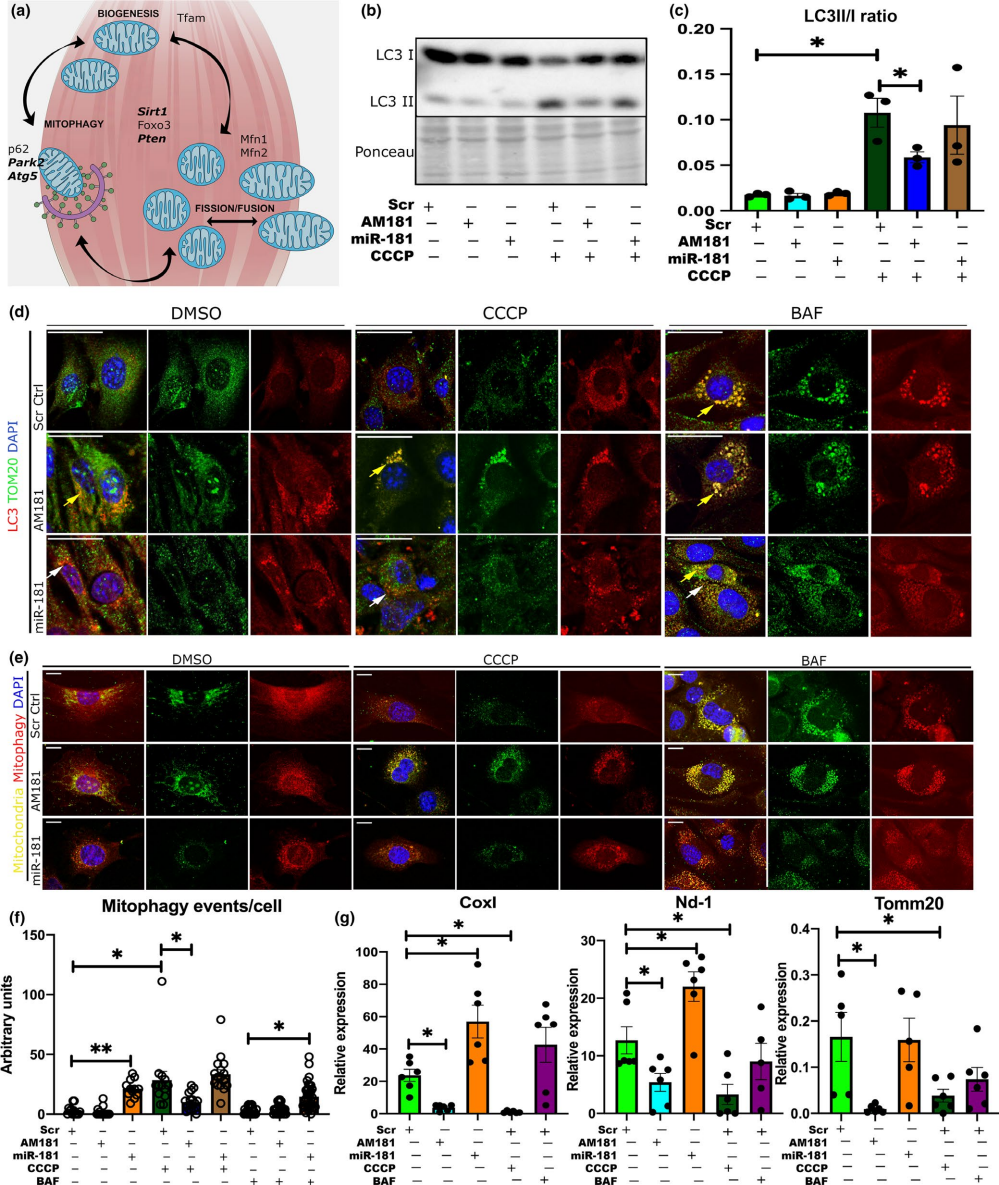
miR‐181a regulates mitochondrial dynamics in vitro. (a) Schematic representation of putative and validated (in bold) miR‐181a targets associated with mitochondrial dynamics. (b, c) LC3 I/II Western blot and quantification of LC3II/I ratio of C2C12 myoblasts following transfections with scrambled antagomiR, miR‐181a mimic, or antagomiR‐181 and CCCP treatment. (d) Immunostaining of C2C12 myoblast transfected with scrambled antagomiR (control), miR‐181a mimic, or antagomiR‐181a for LC3 and the mitochondrial marker TOM20 following DMSO, CCCP, or BAF treatments. Yellow arrows indicate accumulation of LC3B + TOM20+ punctae, and white arrows indicate LC3‐positive punctae. Scale bars show 30 µm. Representative images are shown. (e) Assessment of mitophagic flux in C2C12 cells treated with scrambled antagomiR (control), miR‐181a mimic, or antagomiR‐181a in control (DMSO) or cells treated with CCCP or BAF using mito‐QC construct. Mitochondria are labeled with mCherry and GFP; upon mitophagy induction, GFP signal is quenched. Scale bars indicate 10 µm. (f) Quantification of mitophagic events per cell through analysis of colocalization of red punctae only. (g) qPCR analysis of mitochondrial gene expression (Cox I, Nd‐1, and Tomm20) in C2C12 myoblasts following DMSO, CCCP, and BAF treatments and transfected with scrambled antagomiR (control), miR‐181a mimic, or antagomiR‐181a, respectively, expression relative to β2‐microglobulin shown. Error bars show *SEM*; *p < .05 Student's *t* test

To determine upstream regulators of mitochondrial dynamics, we analyzed genes associated with mitochondrial biogenesis, fission, fusion, and mitophagy for binding sites for microRNAs previously shown to be dysregulated in muscle of mice and humans during aging (Table S1). miR‐181 family was predicted to target multiple genes investigated. miR target prediction databases, TargetScan, miRnet, and miRWalk, identified miR‐181a‐5p (miR‐181a) as a putative regulator of multiple genes associated with mitochondrial dynamics: previously validated targets (highlighted in bold in Figure 3a: Park2, Sirt‐1, PTEN, and Atg‐5, and novel putative targets: p62, DJ‐1, Mfn1, Mfn2, and Tfam (Figure 3a). miR‐181a, and not miR‐181b, miR‐181c, or miR‐181d, was downregulated in TA of mice during aging and exercise of adult mice only (Figure 2g,h). The elevated expression of mitophagy‐associated proteins observed in TA from old mice coupled with a decreased expression of miR‐181a suggested that miR‐181a may act as an important regulator of autophagy and mitochondrial dynamics during muscle aging.

### 2.4 miR‐181a regulates mitochondrial dynamics in myoblasts

To investigate whether miR‐181a regulates mitochondrial dynamics, we used the mitochondrial uncoupler, carbonyl cyanide m‐chlorophenyl hydrazone (CCCP), and autophagic flux inhibitor bafilomycin A1 (BAF) in a C2C12 myoblast model. CCCP treatment decreased expression levels of miR‐181a, while miR‐181a mimic and antagomiR‐181a (AM181a) increased and decreased, respectively, miR‐181a levels in C2C12 cells (Figure S2a). CCCP treatment resulted in formation of LC3 punctae, while BAF treatment resulted in the accumulation of LC3 punctae colocalized with TOM20 (Figure 3d). miR‐181 overexpression resulted in the presence of LC3‐positive punctae in DMSO‐, CCCP‐, and BAF‐treated cells, whereas inhibition of miR‐181a resulted in the accumulation of LC3 and TOM20 colocalized punctae in DMSO, CCCP, and BAF‐treated cells (Figure 3d). Moreover, inhibition of miR‐181a resulted in decreased LC3 II/I in myoblasts treated with CCCP (Figure 3b,c). This suggests inhibition of miR‐181a may result in stalled autophagy.

In order to investigate miR‐181a‐mediated regulation of mitochondrial turnover *via* mitophagy, we used the mito‐QC reporter construct that contains tandem mCherry‐GFP tag fused to the mitochondrial targeting sequence of the outer mitochondrial membrane protein, FIS1 (Allen, Toth, James, & Ganley, 2013). The mitochondrial network fluoresces red and green under normal conditions but during mitophagy, with the delivery of mitochondria to the acidic environment of lysosomes, GFP fluorescence is quenched while mCherry remains stable (Allen et al., 2013; McWilliams et al., 2016). CCCP treatment increased the number of mitochondria associated with lysosomes in Scr controls which returned to basal levels after BAF treatment (Figure 3e,f). AM181a samples showed reduced levels of mitophagic events after CCCP treatment (Figure 3e,f), whereas miR‐181a overexpression led to increased mitophagy events in DMSO and BAF‐treated cells (Figure 3e,f). miR‐181a or AM181a treatment had no effect on cell viability (cytotoxicity assay), and ATP production was mildly increased in myoblasts treated with miR‐181a in the presence of CCCP (Figure S2b).

To investigate the consequences of mitophagy regulation by miR‐181a on mitochondrial content, we analyzed the expression of mitochondrial proteins encoded by the mitochondrial genome: Cox I, Nd‐1 and encoded by the nuclear genome: Tomm20. The expression of all the mitochondrial genes was increased following miR‐181a overexpression and decreased in response to AM181a (Figure 3g).

### 2.5 miR‐181a regulates the expression of p62, Park2, and DJ‐1

**Figure 4:**
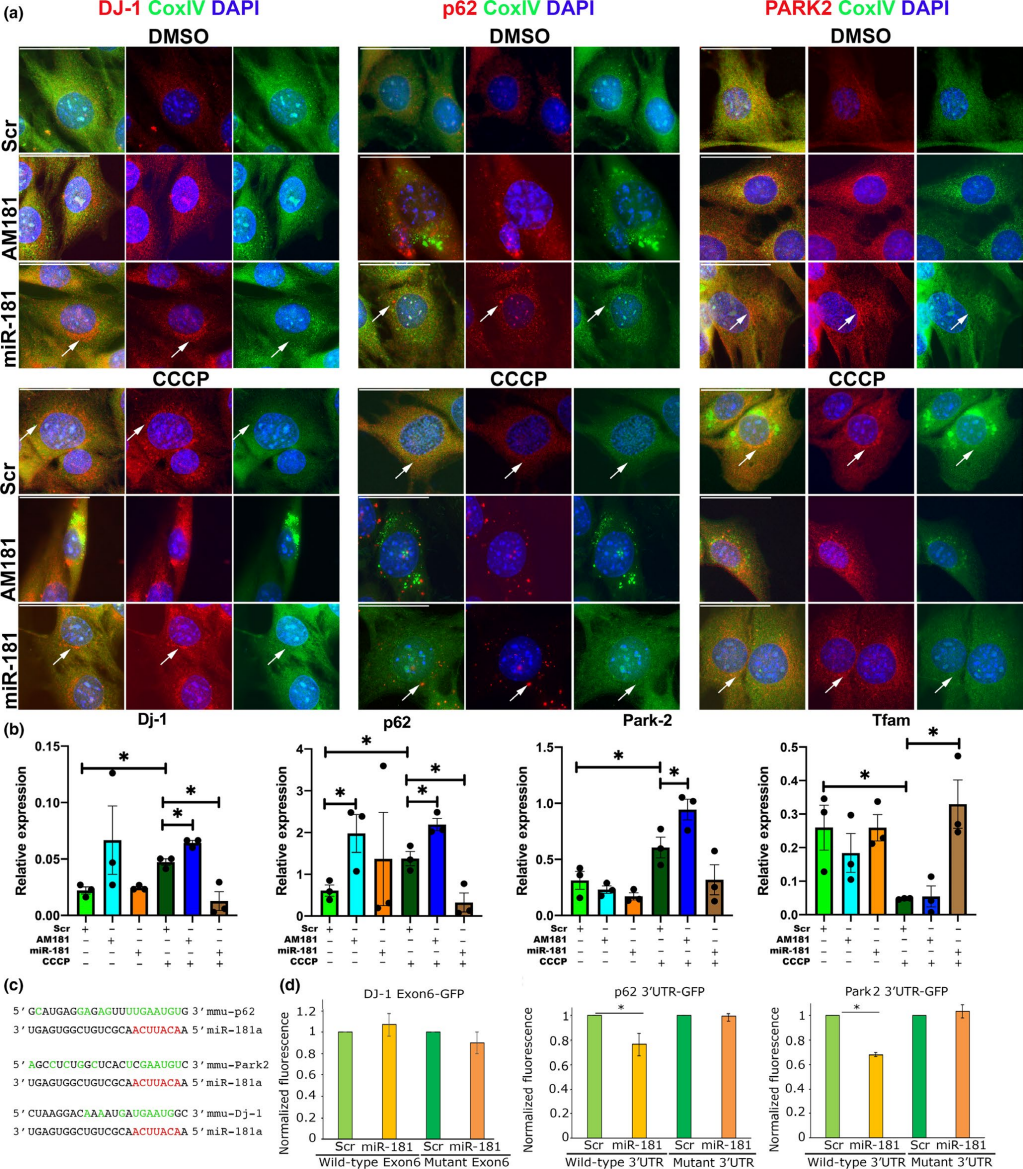
miR‐181a targets p62 and Park2 and DJ‐1 in C2C12 myoblasts. (a) Immunostaining for CoxIV (mitochondrial marker) and mitophagy‐associated genes: p62, DJ‐1, and Park2, showing accumulation of these proteins in C2C12 myoblasts following inhibition of miR‐181a function and turnover of mitochondria following overexpression of miR‐181a. Arrows indicate regions with protein expression and reduced CoxIV expression indicating mitophagy. Representative images are shown. Scale bars indicate 50 µm. (b) qPCR showing the effects of miR‐181 in C2C12 myoblasts following mitophagy induction on the expression of mitophagy‐associated genes, relative to β2‐microglobulin. *n* = 3–4. Error bars show *SEM*. *p < .05; unpaired Student's *t* test. (c) miR‐181a predicted binding sites within the 3′UTRs of murine p62 and Park2 and within an exon of murine DJ‐1. Red—microRNA seed sequence; green—nucleotides complementary to the microRNAs sequence. (d) Normalized GFP fluorescence in myoblasts following co‐transfection with GFP sensor construct containing wild‐type or mutated miR‐181a predicted binding site and miR‐181a mimic or scrambled miRNA mimic. *n* = 3. Error bars show stdev. *p < .05 Student's *t* test

We next validated miR‐181a predicted autophagy‐associated targets in an in vitro model of mitochondrial uncoupling. No changes in the expression of these proteins were detected following miR‐181a overexpression or inhibition in C2C12 myoblasts (Figure S2c,d), possibly due to their high turnover. However, in DMSO‐treated cells, inhibition of miR‐181a resulted in increased levels of p62 mRNA and accumulation of p62 protein which did not colocalize with COXIV mitochondrial marker (Figure 4a,b). miR‐181a overexpression resulted in the presence of p62‐, PARK2‐, and DJ‐1‐positive punctae colocalized with the mitochondrial marker COXIV. In CCCP‐treated cells, inhibition of miR‐181a resulted in accumulation of p62, DJ‐1, and Park‐2 mRNAs and protein with reduced colocalization with COXIV. miR‐181a overexpression resulted in downregulation of the expression of p62, Park2 and DJ‐1 in CCCP‐treated cells to levels comparable to control cells (Figure 4a,b). Concomitant with changes in autophagy‐related genes, miR‐181a overexpression in C2C12 myoblasts treated with CCCP resulted in upregulation of Tfam expression (Figure 4b). These results indicate that miR‐181a increased mitochondrial turnover via mitophagy and potentially concomitant increase in mitochondrial biogenesis as indicated by increase of Tfam expression in C2C12 cells treated with miR‐181. This indicates a role for miR‐181a in maintaining a population of healthy mitochondria within the cells.

We also analyzed changes in localization of BNIP3 and LAMP1 with TOM20 in C2C12 cells treated with miR‐181a or AM181a in control‐, CCCP‐, or BAF‐treated cells. Both experiments demonstrated that AM181a treatment leads to accumulation of BNIP3 or LAMP1 punctae colocalized with TOM20, further demonstrating that low levels of miR‐181a are associated with inhibition of mitophagic flux (Figure S3). However, no miR‐181a binding site could be found in the mRNA sequences of Bnip3 or Lamp1.

To investigate whether miR‐181 directly binds to its target associated with autophagy, we used 3′UTRs of p62 and Park2, and DJ‐1 Exon 6 cloned downstream of GFP reporter constructs. Although miR binding sites within exons are very uncommon, we analyzed this specific putative binding site due to strong effects of miR‐181a on DJ‐1 expression (Figure 4c). GFP reporter constructs were transfected into C2C12 cells in the presence of scrambled (control miR not predicted to bind to 3′UTRs) or miR‐181a mimic. miR‐181a treatment led to significant decrease in GFP fluorescence from GFP‐p62 and GFP‐Park2, but not GFP‐DJ‐1 constructs, as compared to scrambled‐treated controls (Figure 4d). The mutations of miR‐181a binding sites within the 3′UTRs of the investigated genes rendered all constructs nonresponsive to miR‐181a overexpression as compared to scrambled controls. This confirms p62 and Park2 as direct, targets of miR‐181a, whereas DJ‐1 expression may be regulated by miR‐181a in an indirect manner (Figure 4c,d).

### 2.6 miR‐181a regulates expression of p62, DJ‐1, and Parkin in vivo

**Figure 5:**
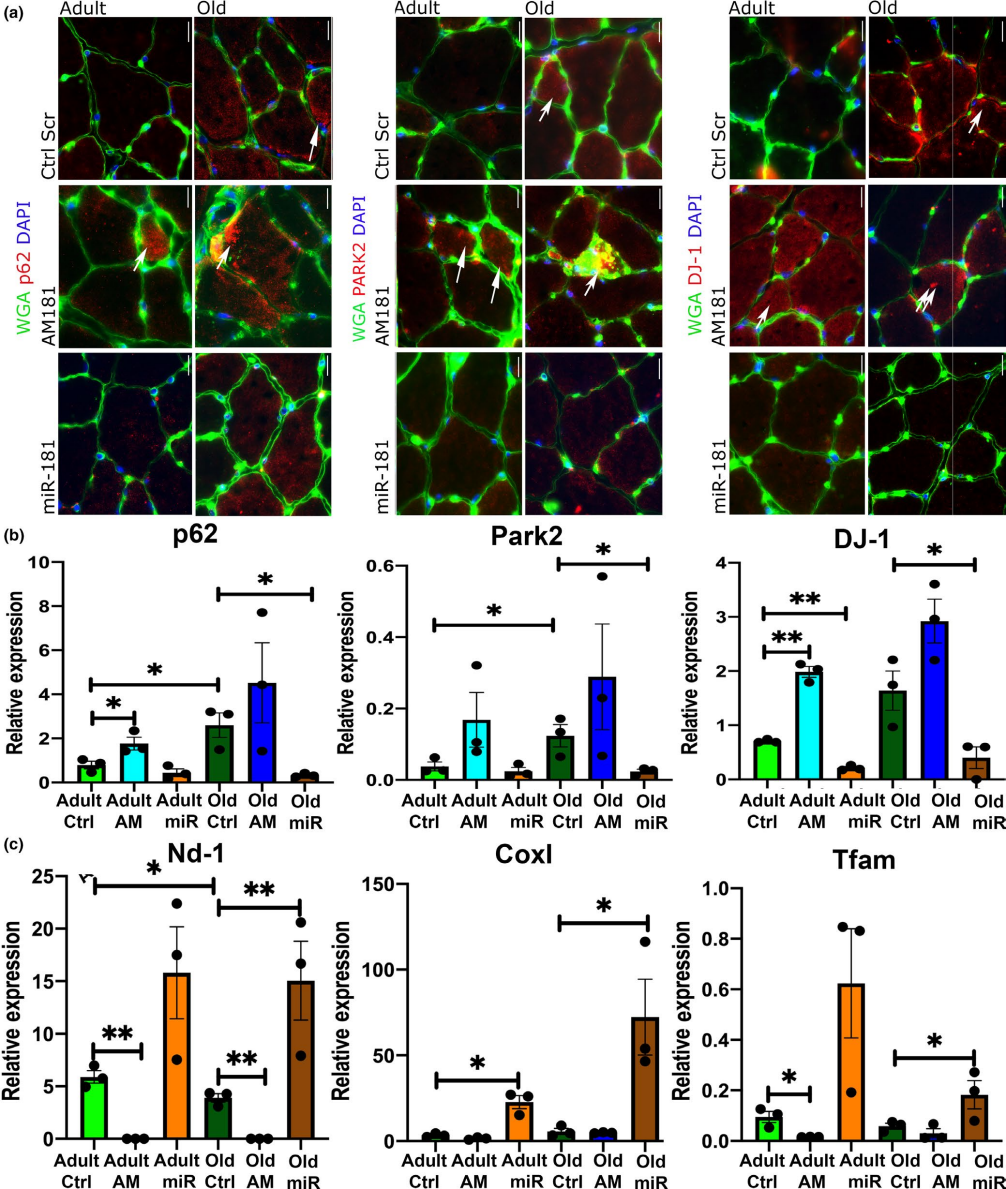
miR‐181a regulates expression of p62, DJ‐1, and Parkin and affects mitochondrial content markers in vivo. (a) Representative images of TAs immunostained for p62, DJ‐1, and Park2 following miR‐181a gain and loss of function. Arrows indicate positive fibers. Scale bars indicate 100 µm. *n* = 3. (b) miR‐181a gain and loss of function in TA of adult and old mice leads to changes in the expression of p62, DJ‐1, and Park2 mRNA, relative to β2‐microglobulin. (c) miR‐181a overexpression increases the expression of mRNA of mitochondrial genes (Cox I, Nd‐1) and regulator of mitochondrial biogenesis (Tfam) in TA of adult and old mice, relative to β2‐microglobulin. Error bars show *SEM* *p < .05 Student's *t* test. Adult—6 months old; old—24 months old male C57BL6/J mice; Ctrl—saline

**Figure 6:**
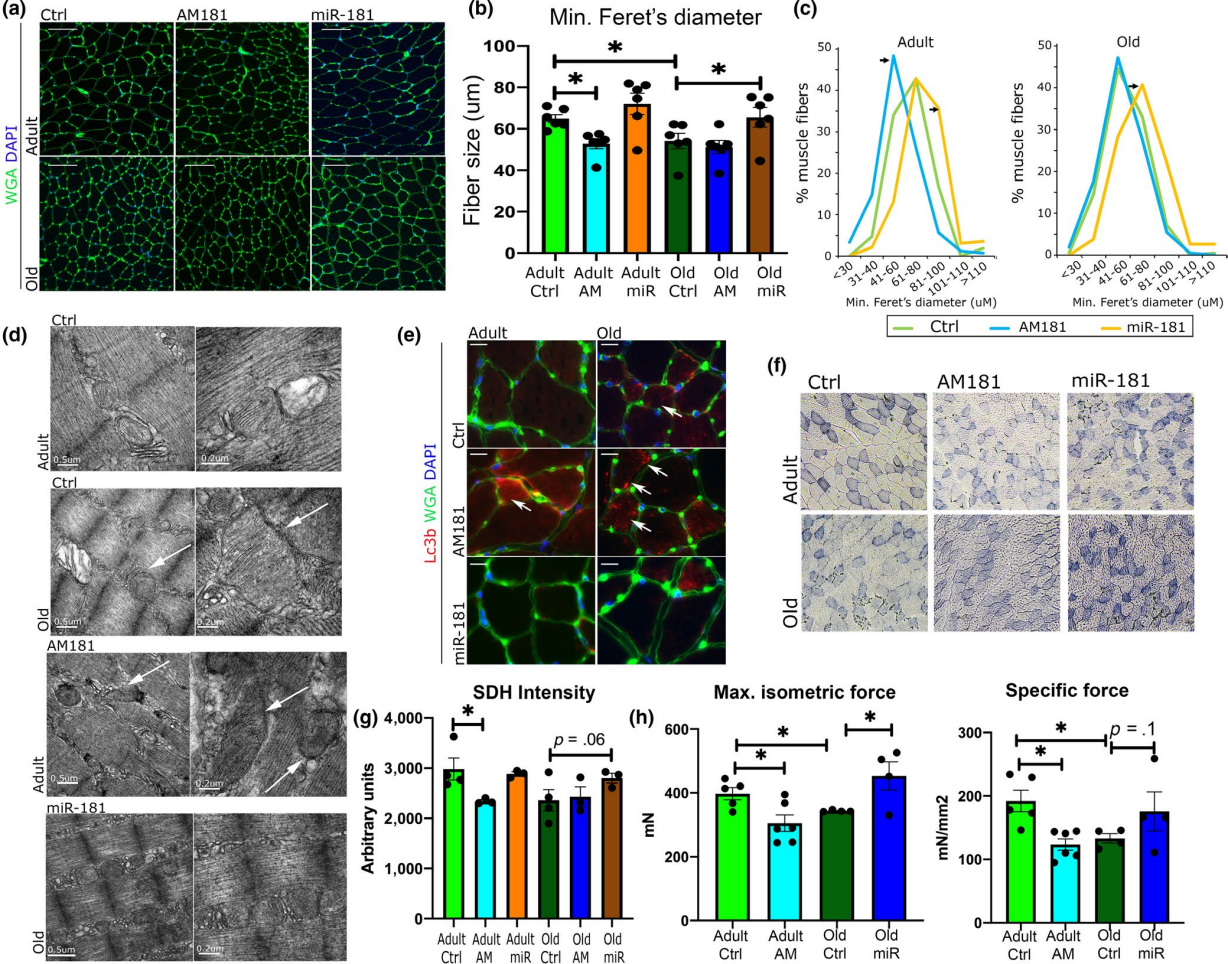
miR‐181a regulates myofiber size and muscle strength in vivo. (a–c) The effects of miR‐181a on myofiber size in adult and old mice. WGA immunostaining (a), quantification of minimal Ferret's diameter (b), and fiber distribution analysis (c) of TA of adult and old mice following intravenous injection of miR‐181a mimic, antagomiR, or saline (control). Scale bars indicate 100 µm; arrows indicate statistically significant changes in fiber distribution (chi‐square test, *p* < .05). *n* = 6; *p < .05 unpaired Student's *t* test. (d) Representative EM images of TA from adult and old mice. Arrows indicate abnormal mitochondria. Scale bars: 0.5 and 0.2 µm. (e) Inhibition of miR‐181a expression in vivo leads to the presence of more of LC3‐positive myofibers (red), *n* = 3; arrows indicate LC3‐positive fibers. (f, g) Histological examination of miR‐181 effects on mitochondria: SDH staining and quantification (f) of TA of adult and old mice following miR‐181 mimic, antagomiR‐181, or saline injections, respectively. Scale bars indicate 200 µm; *n* = 3. (h) Muscle maximum and specific force following miR‐181a gain and loss of function in vivo evaluated in EDL muscles from adult and old mice; error bars indicate *SEM*; *n* = 4–6

The expression and localization of miR‐181a regulated genes were analyzed in the muscle of adult and old mice treated with saline, miR‐181a mimic, or AM181a (Figures 5, 6, and S6). The muscle of control old mice was characterized by the presence of increased p62, DJ‐1, and PARK2‐positive myofibers as compared to the muscle of control adult mice, confirming disrupted or inhibited autophagy during aging (Figure 5a). AM181a treatment of adult mice led to increased number of p62‐, DJ‐1‐, and PARK2‐positive myofibers, whereas fewer p62‐, DJ‐1‐, and PARK2‐positive myofibers were detected in miR‐181a‐treated old mice compared to their respective controls (Figure 5a). Inhibition of miR‐181a in muscle of adult mice led to accumulation of p62 and DJ‐1 mRNA, whereas overexpression of miR‐181a in muscle of old mice led to reduced levels of p62, Park2, and DJ‐1 mRNA as compared to muscle of control old mice to levels observed in muscle of adult mice (Figure 5b). This suggests that lower levels of miR‐181a in muscle are associated with accumulation of its target genes and potentially dysfunctional mitophagy, leading to an accumulation of dysfunctional mitochondria (Figure 6g). To further investigate the role of miR‐181a in regulating mitochondria, we analyzed the expression of mitochondrial genes (CoxI and Nd‐1) in TA muscle of adult and old mice. Consistently, in muscle of old mice, miR‐181a treatment led to increased expression of mitochondrial genes as a marker of mitochondrial content (Figure 5c). Furthermore, we investigated changes in the expression of transcription factors promoting mitochondrial biogenesis: the master regulator of mitochondrial biogenesis (PGC1α) and the mitochondrial transcription factor (Tfam). Consistently in miR‐181a‐treated muscle, the expression of these mitochondrial biogenesis‐associated genes was upregulated (Figures 5c and S5). Together, these results indicate that miR‐181a promotes mitochondrial dynamics through concomitant activation of mitophagy and mitochondrial biogenesis in skeletal muscle from adult and old mice.

### 2.7 miR‐181a regulates mitochondrial quality, myofiber size, and muscle force in vivo

We next investigated the physiological effects of miR‐181a on muscle during aging. Changes in miR‐181a expression in adult and old mice had no significant effect on body or muscle weight (Figure S6). However, miR‐181a inhibition led to a decrease, whereas miR‐181a overexpression led to an increase in myofiber size from adult and old mice, respectively (Figure 6a,b). The relative proportion of fiber sizes within the population of fibers revealed that miR‐181a‐treated mice had a higher proportion of larger fibers, whereas AM181a treatment resulted in a higher population of smaller fibers in muscle from adult mice (Figure 6c). Moreover, a higher proportion of LC3‐positive myofibers was detected in muscle of control old mice and AM181a‐treated adult and old mice, with almost no LC3‐positive fibers detected in adult saline and miR‐181a‐treated mice (Figure 6e). These results indicate miR‐181a‐mediated regulation of mitochondrial content is associated with fiber size.

The relative oxidative potential of muscle as an indication of mitochondrial function was decreased by miR‐181a inhibition in the TA of adult mice, demonstrated by reduced intensity of succinate dehydrogenase (SDH) staining (Figure 6f,g). miR‐181a treatment of old mice resulted in a nonsignificant trend for increased SDH intensity (Figure 6g). EM analysis of mitochondrial populations revealed the presence of swollen and mitochondria with disordered cristae, with some mitochondria expanding longitudinally between myofibrils, in muscle of control old and AM181a‐treated adult mice. miR‐181a treatment of old mice led to a lower proportion of structurally abnormal mitochondria and decreased the abundance of abnormally large mitochondria (Figure 6d).

The physiological relevance of manipulating miR‐181a expression was investigated by examining the effects of miR‐181a on muscle force. Maximum and specific force of the EDL muscle was decreased during aging (Figure 6h). As miR‐181a expression is decreased in muscle during aging, we treated adult mice with AM181a to reduce miR‐181a expression. Conversely, old animals were treated with miR‐181a mimic in an effort to restore miR‐181a expression. Maximum and specific force was decreased in muscle of adult mice treated with AM181a, whereas miR‐181a overexpression in muscle of old mice restored maximum muscle force (Figure 6h). Together, these data suggest that a decrease in miR‐181a expression results in chronic dysregulated mitophagy in muscle during aging associated with presence of abnormal mitochondria, loss of mitochondrial content, and decline of muscle function.

In summary, a decrease in miR‐181a expression resulted in increased expression of autophagy‐associated genes, a decrease in fiber diameter, and specific muscle force in adult mice mimicking aging. Conversely, upregulating miR‐181a levels in old mice improved mitochondrial morphology, increased fiber diameter, and specific force. These data demonstrate microRNA‐mediated fine‐tuning of one of the key mechanisms associated with aging, mitochondrial turnover, and provide proof of principle for the potential use of microRNA‐based therapeutic approaches for aging‐associated disorders such as age‐related muscle atrophy.

## 3 DISCUSSION

In the present study, we have demonstrated decreased mitochondrial content and quality associated with dysregulated expression of autophagy and mitochondrial dynamics‐associated proteins in skeletal muscle during aging. Using bioinformatics and reporter constructs, we have identified miR‐181a as a key regulator of p62 and Park2, and indirectly, regulation of Park7/Protein DJ‐1 expression and localization. Changes in miR‐181a levels and the concomitant changes in the expression of its targets resulted in altered myofiber size, mitochondrial content, and quality and specific muscle force, suggesting miR‐181a may be one of the key regulatory mechanisms underlying age‐related muscle atrophy. This is the first report, to our knowledge, demonstrating a physiologically relevant mechanism of miR‐181a‐mediated fine‐tuning of mitochondrial dynamics on multiple levels through concerted regulation of expression of several autophagy‐associated genes in muscle during aging.

Mitochondrial quality has an important impact on the health and bioenergetics of healthy skeletal muscle and is tightly regulated by mitochondrial biogenesis, fusion, fission, and selective degradation of mitochondria. Mitochondrial content decreases with age in skeletal muscle; however, the exact mechanisms are not fully understood (Hepple, 2014). Functional autophagy and mitophagy are affected by aging and have been associated with changes in mitochondrial dynamics (Carnio et al., 2014; Sebastian et al., 2016). We have detected an increase in the expression of the regulators of Pink1/Parkin‐dependent mitophagy during aging and the presence of abnormal mitochondria, suggesting this mitophagy pathway is disrupted or ineffective, resulting in an accumulation of abnormal mitochondria.

The role of mitophagy and autophagy in skeletal muscle is context‐dependent and even small changes in mitochondrial dynamics can have detrimental consequences on muscle mass and function with an accumulation of abnormal mitochondria (Carnio et al., 2014; Drake, Wilson, & Yan, 2016). Dysregulated mitophagy is associated with decreased functional mitochondria in muscle during aging, where changes in mitochondrial fusion or fission may lead to the presence of abnormally large mitochondria (Mao & Klionsky, 2013). In mice, the deletion of the mitochondrial fusion protein, OPA1, produced a lethal phenotype, while inducible muscle‐specific deletion of OPA1 resulted in a disruption of mitochondrial morphology, proteasome activation, and muscle atrophy (Tezze et al., 2017). Similarly, inducible muscle‐specific deletion of the mitochondrial fission protein, DRP1, resulted in the formation of functionally abnormally large mitochondria, activation of the ER stress response, an increase in Ca^2+^ uptake and myofibre death (Favaro et al., 2019). Interestingly, inducible muscle‐specific deletion of both OPA1 and DRP1 rescued the lethal phenotype of OPA1 knockout, suggesting that in the context of mitochondrial dynamics inhibition of fission has precedence over inhibition of fusion (Romanello et al., 2019).

In *C. elegans*, the age‐related decline in mitophagy inhibits both the removal of dysfunctional mitochondria and impairs mitochondrial biogenesis (Palikaras, Lionaki, & Tavernarakis, 2015). It has been proposed that the age‐related accumulation of dysfunctional mitochondria triggers the activation of SKN‐1, the nematode homologue of Nrf2 (Palikaras et al., 2015). Activation of SKN‐1 triggered a bipartite retrograde signaling pathway coupling the induction of mitochondrial biogenesis and mitophagy genes (Palikaras et al., 2015). It has also been demonstrated that during the myogenic differentiation of C2C12 myoblasts, there is an induction of mitophagy with a corresponding increase in mitochondrial biogenesis (Sin et al., 2016). As miRs have been shown to fine‐tune gene expression, they are ideal candidates to control this delicate between removal of dysfunctional/damaged mitochondria and mitochondrial biogenesis. Several microRNAs, including miR‐149, miR‐761, and miR‐494, have been identified that can inhibit or promote mitochondrial biogenesis (Mohamed, Hajira, Pardo, & Boriek, 2014; Xu, Zhao, Sun, Liu, & Zhang, 2015; Yamamoto et al., 2012) for review (Dahlmans, Houzelle, Schrauwen, & Hoeks, 2016).

In this study, we identified miR‐181a, which has decreased expression in muscle during aging, as a putative regulator of multiple genes associated with the regulation of mitochondrial dynamics. miR‐181a‐mediated regulation of components of autophagy has previously been reported in aging, cancer cell lines, and nervous system (Cheng et al., 2016; Indrieri et al., 2019; Rippo et al., 2014; Soriano‐Arroquia, House, Tregilgas, Canty‐Laird, & Goljanek‐Whysall, 2016; Tekirdag, Korkmaz, Ozturk, Agami, & Gozuacik, 2013). Recently, overexpression of miR‐181a/b‐1 in chondrocytes resulted in improvements in mitochondrial metabolism (Zheng, Liu, Tycksen, Nunley, & McAlinden, 2019). Our results indicate miR‐181a regulates autophagy/mitophagy and effector proteins, providing a mechanism of fine‐tuning mitochondrial dynamics through parallel regulatory pathways. We have demonstrated that miR‐181a regulates the expression and localization/function of multiple autophagy‐related genes: Park2, p62, and Protein DJ‐1/Park7 (Figures 3, 4 and 5), concomitant with upregulation of mitochondrial biogenesis. Mitophagy and mitochondrial biogenesis have been demonstrated to be coupled previously (Palikaras et al., 2015; Sin et al., 2016). Moreover, restored expression of miR‐181a in the muscle of old mice prevented accumulation of mitophagy‐associated proteins and was related with increased mitochondrial content and improved mitochondrial quality (Figures 5 and 6). We also observed a decrease in the presence of swollen and abnormally large mitochondria in muscle of old mice following miR‐181a overexpression (Figure 6). Although these changes could be associated with miR‐181a predicted target genes: Mfn1 and Mfn2, we did not observe consistent regulation of these genes by miR‐181a at the mRNA level (Figures S1 and S5). Finally, we have shown that miR‐181a regulates myofiber size and muscle force: Adult and old mice treated with AM181a or miR‐181a had significant changes in the expression of genes linked with mitochondrial dynamics, mitochondrial quality, and muscle fiber diameter and specific force of muscle (Figure 6).

In summary, we propose that miR‐181a regulates skeletal muscle homeostasis and muscle metabolism, size, and force, by regulating mitochondrial dynamics. miR‐181a is downregulated during aging, and we propose that concomitant upregulation and accumulation of its mitophagy‐associated targets paradoxically lead to a stalling or inhibition of mitophagy, resulting in the accumulation of damaged mitochondria. We have demonstrated that fine‐tuning the levels of autophagy regulatory (p62) and mitophagy‐associated proteins (DJ‐1, Park2) and concomitant changes in the expression of Tfam, by overexpressing miR‐181a, leads to improved mitochondrial dynamics resulting in increased myofiber size and muscle function. In this study, we focused on the predicted and confirmed targets of miR‐181a many of which are associated with the Pink/Parkin mitophagy pathway. It was therefore surprising to find that regulation of Parkin, p62, and Protein DJ‐1 by miR‐181a overexpression resulted in increased mitophagy and mitochondrial biogenesis. Functional experiments in vitro and in vivo demonstrated that decreased miR‐181a resulted in the accumulation of these target proteins. Low levels of miR‐181a in aging muscle were associated with accumulation of its target proteins and stalled autophagy. Our data suggest that miR‐181a regulates mitochondrial dynamics by fine‐tuning the Pink/Park pathway and mitochondrial biogenesis, potentially in response to stress factors. Moreover, mitophagy is coordinated by a number of conserved cellular pathways, and disruption of this balance can result in the accumulation of dysfunctional mitochondria and aging (Palikaras et al., 2018). Exercise increases mitochondrial biogenesis in skeletal muscle, and the increased expression of miR‐181a in response to acute exercise (this study and (Safdar, Abadi, Akhtar, Hettinga, & Tarnopolsky, 2009) suggests that miR‐181a plays a key role in improving overall mitochondrial health. Our results indicate overexpression of miR‐181a increases the expression of genes required for mitochondrial biogenesis and promotes mitophagy either directly or indirectly to restore mitochondrial dynamics. There are a number of limitations to the work presented in this manuscript. We have determined from in vitro studies that miR‐181a promotes mitophagy,however, mitophagic flux was not directly assessed in animal models. Similarly, we used microscopy and the expression levels of PGC1α, Tfam, and mitochondrial encoded genes Nd‐1 and Cox‐1 as indirect measures of mitochondrial biogenesis in our models. Furthermore, miR‐181a overexpression resulted in improved physiological parameters in old mice, but the effects on mitochondrial dynamics in other tissues were not examined. We also only analyzed male mice in this study and any future work both male and female mice should be included.

Despite these limitations, the results of this study demonstrate for the first time the microRNA‐mediated regulation of mitochondrial content in muscle during aging with physiologically relevant consequences on myofiber size and strength indicating the potential of microRNA‐based therapies for age‐related muscle atrophy.

## 4 EXPERIMENTAL PROCEDURES

### 4.1 Mice

The study was performed using male wild‐type C57Bl/6 mice (adult: 6 months old; old—22–24 months old at the beginning of the treatment). Mice were obtained from Charles River (Margate). All mice were maintained under specific pathogen‐free conditions and fed ad libitum a standard chow and maintained under barrier on a 12‐hr light–dark cycle. For miR‐181a‐5p expression manipulation, mice were injected with 2mg/kg body weight three times during 4‐week period with miR‐181a mimic (GE Healthcare, C‐310435‐05 conjugated to cholesterol) or custom antagomiR‐181a (5′‐FITC‐ mA*mC*mUmCmAmCmCmGmAmCmAmGmCmGmUmUmGmAmA*mT*mG*mU*mU—3′Cholesterol; m—hydroxymethyl modified bases; *—phosphorothioate bonds; CH—cholesterol) or saline (control mice). *Extensor digitorum longus* (EDL) muscle force measurements and tissue collection were performed four weeks from the first injection. For tissue collection, mice were culled by cervical dislocation. The tissues were immediately excised, portions of TA muscles were either directly frozen and stored at −80°C for qPCR or homogenised for proteomics and western blotting. A portion of the TA muscle was also embedded in OCT, frozen is isopentane and stored at −80°C. Experiments were performed in accordance with UK Home Office guidelines under the UK Animals (Scientific Procedures) Act 1986 and received ethical approval from the University of Liverpool Animal Welfare and Ethical Review Body. For each experiment, *n* = 3 (Western blot and qPCR analyses) or *n* = 6 (adult mice) or *n* = 4 (old mice; force measurements) mice were used.

### 4.2 Cell culture

C2C12 myoblasts were maintained in DMEM (Sigma‐Aldrich) supplemented with 10% fetal bovine serum and 1% penicillin/streptomycin (Sigma‐Aldrich; Goljanek‐Whysall et al., 2012). For cholesterol‐linked miR‐181a mimic, antagomiR‐181a, or antagomiR scrambled, 50% confluent C2C2 myoblasts were treated at 100 nM final concentration for 48 hr previous to treatment with 10 µM CCCP or 100 nM bafilomycin A (both Sigma‐Aldrich). 16 hr following CCCP treatment or 1 hr after bafilomycin A treatment, RNA and protein were isolated from treated cells or C2C12 cells were fixed in ice‐cold methanol and immunostained as described below (muscle staining). For mitophagy flux analyses, mito‐Qc reporter was used as previously described (Allen et al., 2013). In brief, mito‐QC viral molecules produced in HEK293T cells were used to transduce proliferating C2C12 cells. C2C12 cells were simultaneously treated with miR‐181a mimic or antagomiRs and treated with DMSO (control), CCCP, or bafilomycin A and imaged as described above. Images were obtained either using a Zeiss fluorescent upright microscope (Model Imager M1) couples with AxioCam MRm and AxioVision software 4.8 or with an Olympus Fluoview3000 Laser confocal microscope and analysed using ImageJ as described previously (Soriano‐Arroquia, McCormick, Molloy, McArdle, & Goljanek‐Whysall, 2016). Cytotoxicity and ATP generation were measured using mitochondrial ToxGlo assay (G8000) from Promega as per the manufacturer's instructions.

### 4.3 In situ muscle function analysis

Force measurements of the EDL muscles were performed as described before via peroneal nerve stimulation in adult and old male mice (Sakellariou et al., 2016). Briefly, mice were anesthetized using ketaset and dormitor. The distal tendon was secured to the lever arm of a servomotor (Aurora Scientific). Muscle optimal length (*L*_o_) was determined with during a series of 1 Hz stimulation and set at the length that generated the maximal force. EDL muscles were electrically stimulated to contract at *L*_o_ and optimal stimulation voltage (10 V) at 2 min intervals for 300 ms with 0.2 ms pulse width to determine maximum isometric tetanic force (*P*_o_). The frequency of stimulation was increased from 10 to a maximum of 300 Hz in 50 Hz increments. *P*_o_ was identified when the maximum force reached a plateau. Muscle fiber length (*L*_f_) and weight of EDL muscles were measured ex vivo, and muscle cross‐sectional area (CSA) was calculated. Specific *P*_o_ (mN/mm^2^) was calculated by dividing *P*_o_ by total fiber CSA.

### 4.4 Muscle staining

TA muscles were cryosectioned at −20°C through the mid‐belly. 10‐μm sections were rinsed with phosphate‐buffered saline (PBS) and fixed in ice‐cold methanol for 5 min. Sections were incubated in blocking solution (1% BSA: Sigma‐Aldrich, 10% normal horse serum: Thermo Fisher Scientific, UK 0.3 M glycine: Sigma‐Aldrich, Dorset UK, in 0.1% PBS‐Tween: Sigma‐Aldrich) for 1 hr at room temperature and next incubated with primary antibodies SQSTM1/p62: Abcam, UK, ab109012 (anti‐rabbit), CoxIV: Cell Signaling, 3E11, Tomm20: Abcam, UK, ab56783 (anti‐mouse) or ab186734 (anti‐rabbit), Lc3b: Abcam, UK, ab10912, DJ‐1: Abcam, UK, ab76241, Parkin: Abcam, UK, ab15954, Pink1: Abcam, UK, ab23707 (all 1:1,000 dilution in blocking buffer) diluted in 5% horse serum, 0.01% Triton X in PBS for 2 hr at room temperature. Three PBS washes were performed, and sections were next incubated with secondary antibodies diluted in 5% horse serum, 0.01% Triton X in PBS (anti‐rabbit‐Alexa 647, anti‐mouse—Alexa 532 or 488, Thermo Scientific). Fluorescein‐labeled wheat germ agglutinin (WGA, Vector Laboratories, 5 μg/ml) was used to identify extracellular matrix. Nuclei were identified using 4′,6‐diamidino‐2‐phenylindole (DAPI, 1 μg/ml, Sigma‐Aldrich). SDH staining was performed as previously described (Smith et al., 2018). Transverse sections from 3 to 4 muscles/treatment group were examined by blinded observers to count the minimal Feret's diameter. ImageJ software was used to analyze individual muscle fibers as described previously (Sakellariou et al., 2016).

### 4.5 Proteomics and pathway analysis

TA muscles were immediately dissected and a portion homogenized directly in 50 mM ammonium bicarbonate pH 8. Protein lysates were prepared by centrifugation at 15,000 *g* for 10 min at 4°C, and protein concentrations were calculated using Bradford assay (Bio‐Rad) with BSA as a standard. 100 µg of protein extract was diluted to 160 µl with 25 mM ammonium bicarbonate and denatured by the addition of 10 µl of 1% RapiGest (Waters) in 25 mM ammonium bicarbonate and incubated at 80°C for 10 min with shaking. 10 µl of a 100 mM solution of Tris(2‐carboxyethyl)phosphine hydrochloride (TCEP) was added to reduce reversibly oxidized Cys residues followed by incubation at 60°C for 10 min. Cys was then alkylated by addition of N‐ethylmaleimide and incubated at room temperature for 30 min. An aliquot of the samples was used at this point to check procedure by SDS‐PAGE. Proteolytic digestion was performed by addition of Trypsin (Thermo Scientific, Paisley, UK) followed by overnight incubation at 37°C. Digestion was terminated and RapiGest removed by acidification (3 µl of TFA and incubated at 37°C for 45 min) and centrifugation (15,000 *g* for 15 min).

#### 4.5.1 LC–MS/MS and label‐free MS quantification

Samples were analyzed using an Ultimate 3000 RSLC nano system (Thermo Scientific) coupled to a QExactive mass spectrometer (Thermo Scientific). 2µl of sample was diluted in 18 µl buffer (97% H_2_O, 3% MeCN and 0.1% formic acid v/v), and 5 µl corresponding to 250 ng of protein was loaded onto the trapping column (PepMap 100, C18, 75 µm × 20 mm) using partial loop injection for 7 min at a flow rate of 4 µl/min with 0.1% (v/v) TFA. Sample was resolved on the analytical column (Easy‐Spray C18 75 µm × 400 mm, 2 µm column) using gradient of 97% A (0.1% formic acid) and 3% B (99.9% ACN and 0.1% formic acid) to 60% A and 40% B over 120 min at a flow rate of 300 nl/min. Data‐dependent acquisition consisted of a 70,000 resolution full‐scan MS scan (AGC set to 10^6^ ions with a maximum fill time of 250 ms), and the 10 most abundant peaks were selected for MS/MS using a 17,000 resolution scan (AGC set to 5 × 10^4^ ions with a maximum fill time of 250 ms) with an ion selection window of 3 *m/z* and normalized collision energy of 30. Repeated selection of peptides for MS/MS was avoided by a 30‐s dynamic exclusion window.

Label‐free relative quantification was performed using PEAKS7 software (Bioinformatics Solutions Inc.) and was searched against the UniProt mouse sequence database using an in house Mascot server (Matrix Science). Search parameters used were peptide mass tolerance 10 ppm; fragment mass tolerance 0.01 Da, 1+, 2+, 3+ ions; missed cleavages 1; and instrument types ESI‐TRAP. Variable modifications included in search were oxidation of methionine and cysteine residues and N‐ethylmaleimide on cysteines. The mass spectrometry proteomic data have been deposited to the ProteomeXchange Consortium via the PRIDE (Vizcaino et al., 2016) partner repository with the dataset identifier PXD01009. Label‐free quantification data were imported into Perseus (Tyanova et al., 2016) software for generation of heatmaps. Heatmaps represent proteins that significantly change (>2 fold change in abundance −10log*P*‐value > 20 (equivalent to a *p*‐value < .01) and identified with a false discovery rate of < 1%. Supporting information including the label free proteomic data has been deposited into the Mendeley data set https://doi.org/10.17632/gksmyhy89r.1. Pathway analysis of label‐free quantitative proteomic data was performed using PathVisio (Kutmon et al., 2015) to visualize and highlight altered pathways from detected proteins.

### 4.6 Real‐time PCR and Western blotting

cDNA synthesis (mRNA) was performed using 500 ng RNA and SuperScript II (Thermo Scientific), and cDNA synthesis (microRNA) was performed using 100 ng RNA and miRscript RT kit II (Qiagen) according to the manufacturer's protocol. qPCR analysis was performed using miScript SYBR Green Master Mix (Qiagen) or sso‐Advanced SYBR Green Master Mix (Bio‐Rad) in a 10 µl reaction. Expression relative to β2‐microblobulin or Rnu‐6 and/or Snord‐61 (microRNA) was calculated using delta–delta Ct method. *p*‐value was calculated using unpaired Student's *t* test. The sequences of primers used are included in Table S2. For Western blotting, homogenized protein lysates were diluted using Laemmli buffer and separated on 12% SDS‐PAGE gels. Briefly, 20 µg of protein was loaded, proteins were transferred using a semi‐dry blotter, and after transfer, membranes were stained with Ponceau S to ensure equivalent loading. Membranes were blocked in 3% milk in TBS‐T, and following washing in TBS‐T, membranes were incubated with primary antibodies (as above) at a dilution of 1 in 1,000 in blocking buffer. Goat anti‐rabbit HRP secondary antibody (Cell Signaling) was diluted 1 in 3,000 in TBS‐T. Thermo super signal west dura was used for chemiluminescence detection using a Chemidoc (Bio‐Rad), and images were acquired and analyzed using Image Lab 5.0 software (Bio‐Rad) and normalized using Ponceau S stain.

### 4.7 In vitro miRNA target prediction and validation

miR‐181 targets were predicted using TargetScan v.6.2 (http://www.targetscan.org/, release June 2012) and miRWalk (http://zmf.umm.uni-heidelberg.de/apps/zmf/mirwalk2/) using mouse and human searches and broadly conserved microRNA target sites settings. 3′UTR regions with wild‐type or mutated miR‐181a target site were synthesized using GeneArt service (Thermo Scientific) and cloned into a GFP TOPO vector (Thermo Scientific). Sequences cloned are listed in Table S3. C2C12 myoblasts were cultured in 96‐well plates and transfected using Lipofectamine 2000™ (Thermo Scientific) with WT or mutant sensor (200 ng), with 100 nM miR scrambled or miR‐181a mimic (100 nM, GE Healthcare; Soriano‐Arroquia, House, et al., 2016). Each experiment was carried out using at least two independent plasmid preparations in triplicates. GFP fluorescence was measured 48 hr following transfections using FLUOstar Optima microplate reader (BMG Labtech).

### 4.8 Quantification and statistical analysis

#### 4.8.1 Statistical analysis of SDH staining, myofiber size, and muscle force

All data are represented as mean ± *SEM*. Data normality was assessed, and unpaired Student's *t* test or chi‐square test, as indicated in figures, was performed using Prism version 5.01 software package for Windows (GraphPad Software, http://www.graphpad.com). *p*‐value < .05 was considered as statistically significant, *n* = 3–6 as indicated in figure legends.

#### 4.8.2 qPCR data

Expression relative to β2‐microblobulin or Rnu‐6 and/or Snord‐61 (microRNA) was calculated using delta–delta Ct method. *p*‐value was calculated using unpaired Student's *t* test.

#### 4.8.3 Western blotting

Band intensities were normalized using overall total protein intensity from Ponceau S staining. Relative expression was calculated using unpaired Student's *t* test with *p*‐value < .05 considered significant.

#### 4.8.4 Label‐free proteomics

Proteins were quantified using top3 method using PEAKS7 label‐free software, and proteins were considered significantly changed with *p*‐value < .01 and >2 fold change.

## CONFLICT OF INTEREST

The authors declare they have no conflict of interest.

## AUTHOR CONTRIBUTIONS

KGW and BMD conceptualized the study. KGW and BMD contributed to methodology. ASA, RMC, CC, KGW, and BMD carried out investigation. KGW and BMD wrote the original draft. KGW, BMD, RMC, and ASA wrote, reviewed, and edited the manuscript. KGW acquired funding. KGW and BMD supervised the study.

## Supporting information

Supplementary Material

## Data Availability

The mass spectrometry proteomic data have been deposited to the ProteomeXchange Consortium via the PRIDE (Vizcaino et al., 2016) partner repository with the dataset identifier PXD01009. Source data for Western blotting, qPCR primer sequences, sequences of 3′UTR cloned into GFP reporter construct sequences, and label‐free proteomic data have been deposited into Mendeley data set https://doi.org/10.17632/gksmyhy89r.1.
