# Supplementary material for: miR‐181a regulates p62/SQSTM1, parkin, and protein DJ‐1 promoting mitochondrial dynamics in skeletal muscle aging
Source: Aging Cell. 2020 Apr 15;19(4):e13140. doi: 10.1111/acel.13140 (PMC7189996; doi:10.1111/acel.13140)
Supplement: Supplementary file 1 — Supplementary Material [file ACEL-19-e13140-s001.docx]

**Supplementary Files**

**
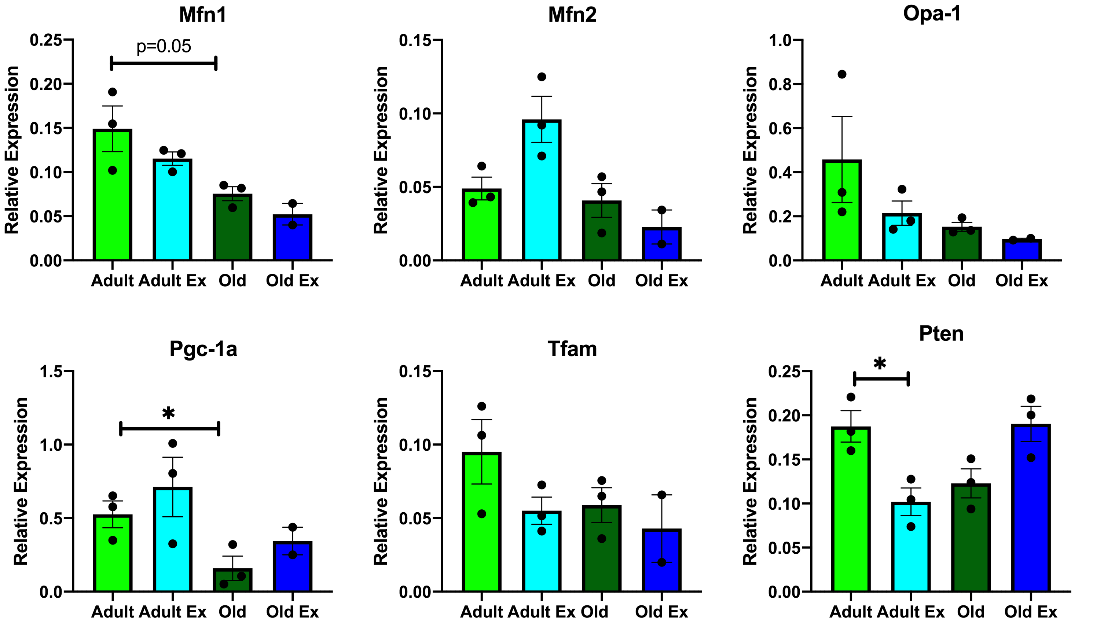
**

**Figure S1.** Expression of mitochondrial dynamics-associated genes in TA muscle of adult and old mice. Adult – 6 months old; old – 24 months old male C57BL6/J mice. Ex – TA following isometric contraction protocol. Expression relative to β2-microglobulin is shown. Representative images are shown. n=3. Error bars show SEM. * - p<0.05 Student’s t-test.


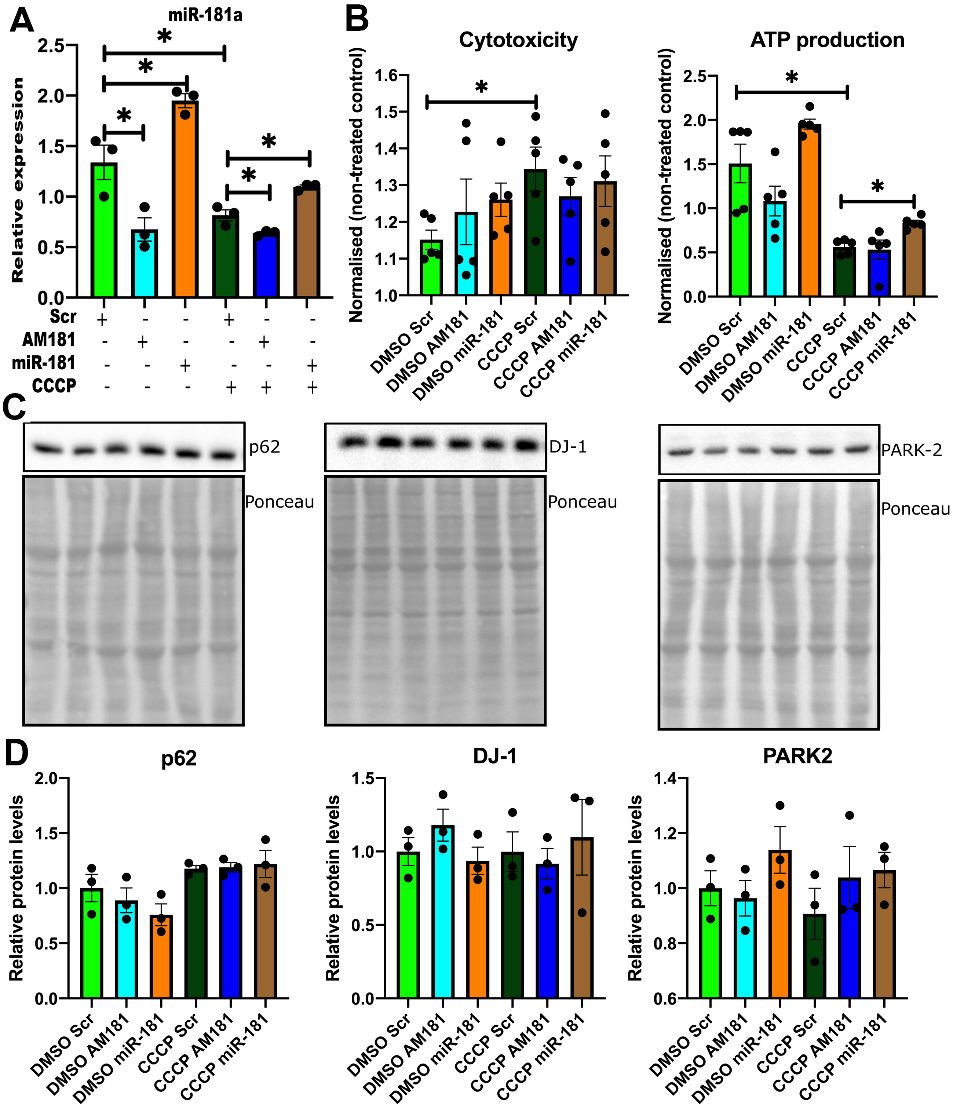


**Figure S2. miR-181 does not regulate myoblast viability.**

**A.** qPCR of miR-181a expression in C2C12 myoblasts following 10 µM CCCP treatment and transfections with scrambled antagomiR (control), miR-181a mimic or antagomiR-181a, respectively, relative to Rnu-6 expression.

**B**. C2C12 myoblasts treated with miR-181 mimic or anyagomiR-181 do not show decreased viability, however miR-181a treatment may improve mitochondrial function in the presence of CCCP, as compared to control. Mitochondrial ToxGlo assay (Promega) used.

**C. D.** Western blot and quantification of p62, DJ-1 and PARK-2 in C2C12 myoblasts treated with miR-181a mimic, antagomiR or control antagomiR (Scr) in control (DMSO) conditions or with CCCP treatment; representative western blots are shown.

*: p<0.05, unpaired Student T test. Error bars show SEM; n=3.


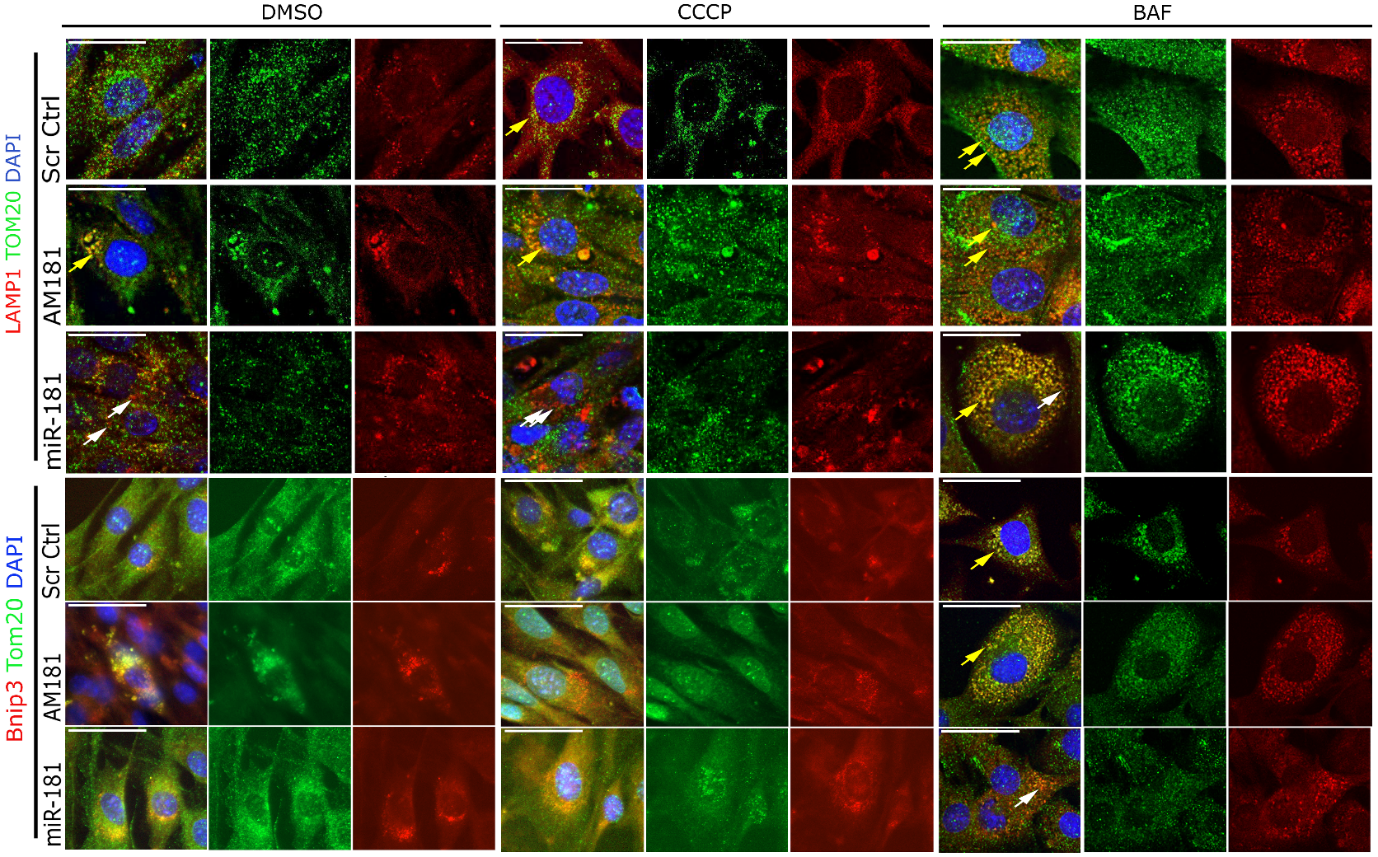


**Figure S3**. Immunostaining for TOM20 (mitochondrial marker) and mitophagy-associated proteins: LAMP1 and BNIP3 in C2C12 myoblasts following inhibition or overexpression of miR-181a in control (DMSO) or conditions promoting (CCCP) or inhibiting (BAF – bafilomycin) autophagy. Representative images are shown.


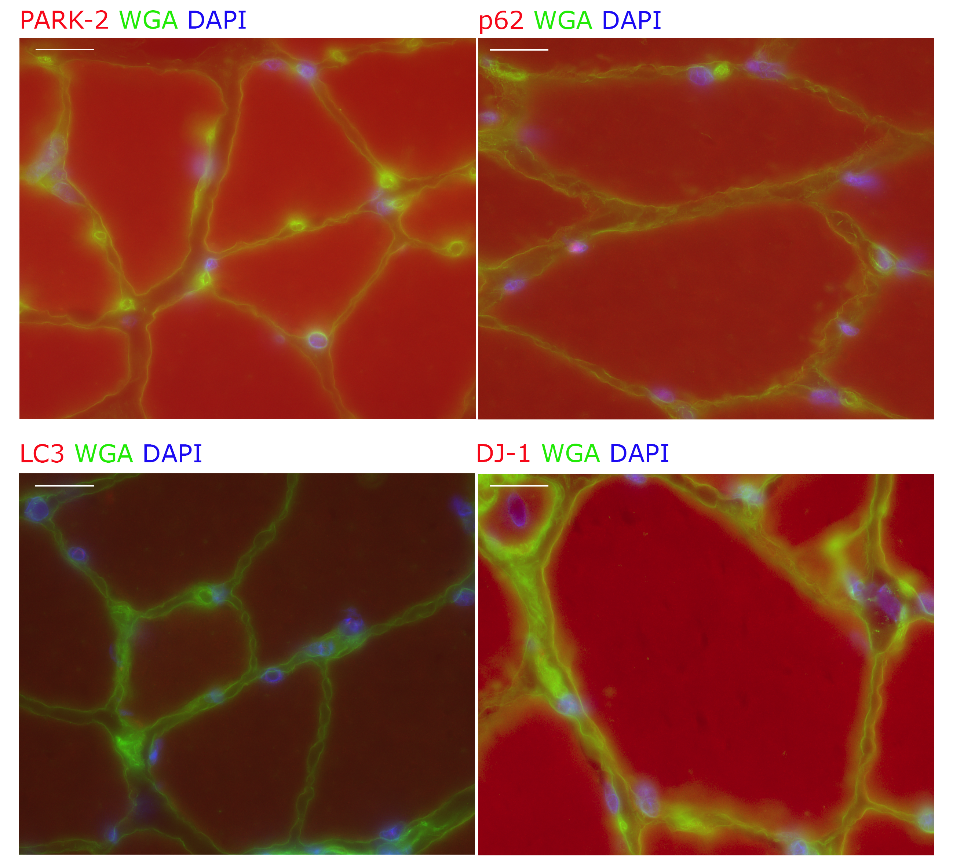


**Figure S4**. Negative control for immunostaining. Representative images of TAs negative controls (no primary antibody) immunostained for p62, DJ-1 and Park2 following miR-181a gain- and loss-of-function. Scale bars indicate 100 µm.

**
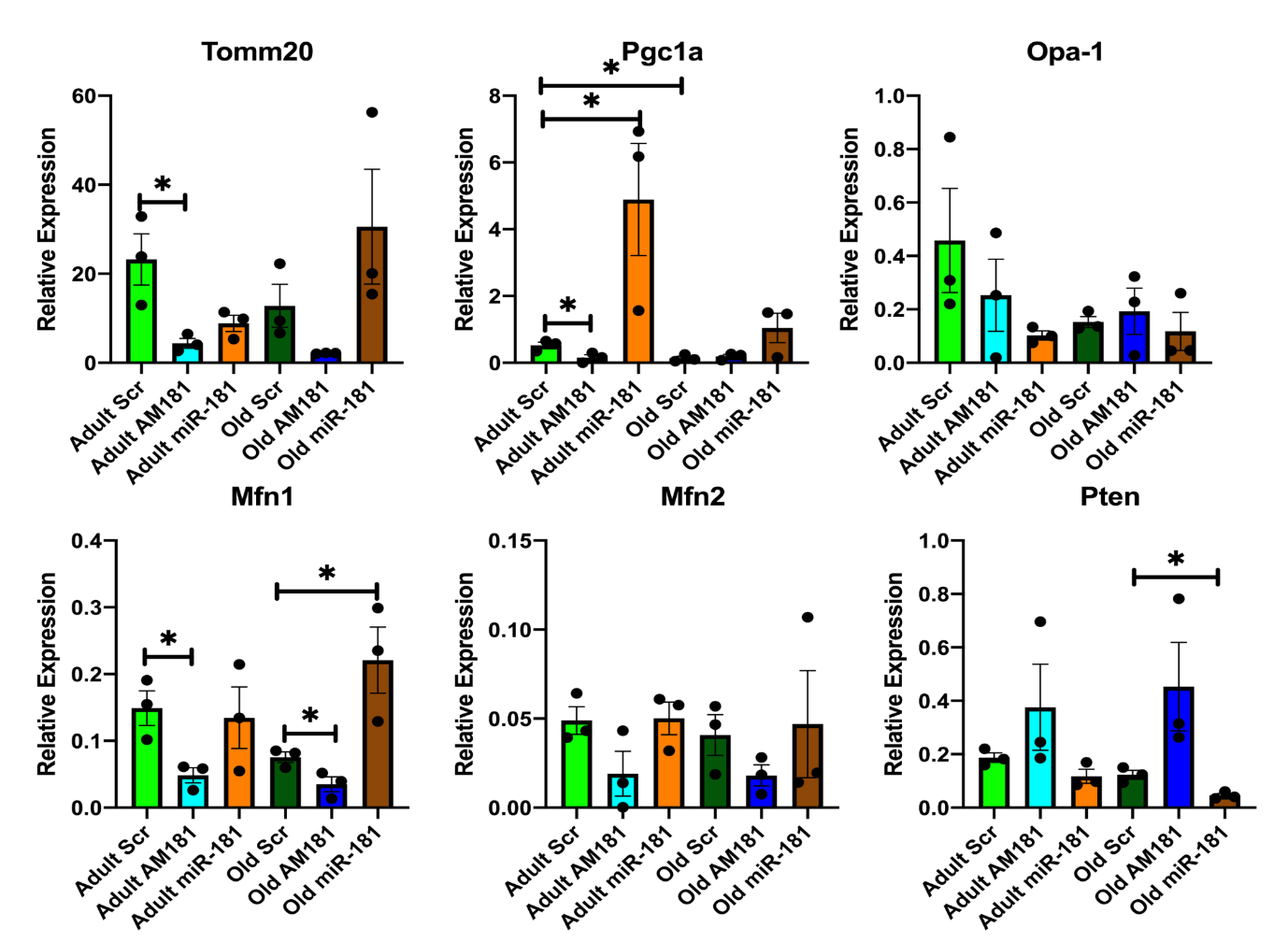
**

**Figure S5**. miR-181a gain- and loss-of-function in TA of adult and old mice leads to changes in the expression of several but not all mitochondrial dynamics-associated genes, relative to β2-microglobulin. Error bars show SEM * - p<0.05 Student T-test. Adult – 6 months old; old –24 months old male C57BL6/J mice; Scr - saline.


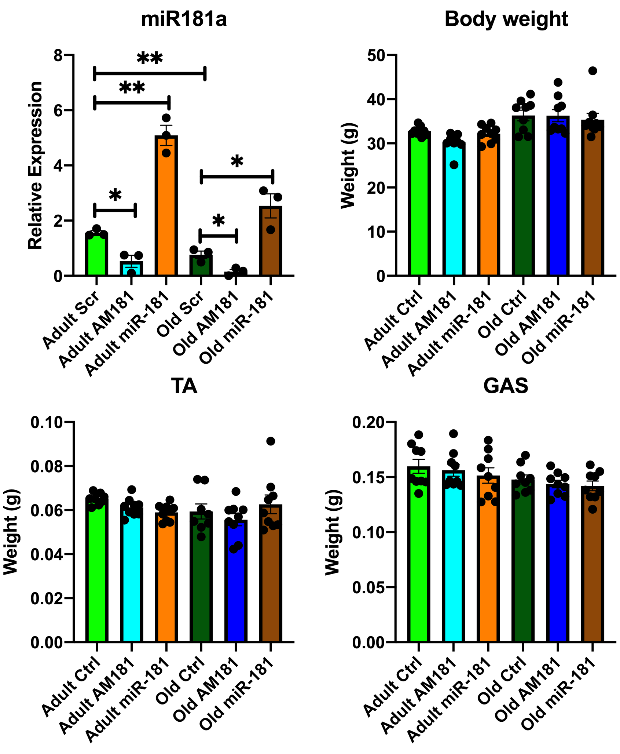


**Figure S6**. miR-181a mimic and antagomiR are effectively delivered into TA muscle via intravenous injections, however do not affect body weight or muscle mass. Changes in miR-181a expression in TA muscle of adult and old mice following intravenous injections of miR-181a mimic or antagomiR181a as compared to saline injected mice were detected by qPCR. Expression relative to Rnu-6. Error bars show SEM * - p<0.05 Student T-test. Adult – 6 months old; old –24 months old male C57BL6/J mice; Scr - saline.

Table S1. microRNAs, levels of which are dysregulated in muscle of mice and humans during ageing, based on Soriano et al., Drummond et al., Rivas et al.

| microRNA expression of which changes in muscle during ageing. | Predicted/validated targets associated with mitochondrial biogenesis, fusion, fission, mitophagy or autophagy |
| --- | --- |
| miR-181-5p | P62, Tfam, Mfn1, Mfn2, Sirt1, Park2, DJ-1, Atg5, Pten, |
| miR-379-5p | Mfn1, Park2, |
| miR-26a-5p | Tfam, Park2, Lc3b, Pten, Ulk2, Mff, |
| miR-30c-5p | Pgc1a, Sirt1, Foxo3, Bnip3l, Atg5, |
| miR-499-5p | Drp1, Park2, Bnip3, Pten, |
| miR-208-3p | Drp1, |
| miR-133a-3p | Sirt1, Foxo3, Lamp1, Bnip3l, Mid49 |

References for Table S1:

Drummond MJ, McCarthy JJ, Sinha M, Spratt HM, Volpi E, Esser KA, Rasmussen BB (2011b) Aging and microRNA expression in human skeletal muscle: a microarray and bioinformatics analysis. Physiol Genomics 43(10):595–603

Rivas DA, Lessard SJ, Rice NP, Lustgarten MS, So K, Goodyear LJ, Parnell LD, Fielding RA (2014a) Diminished skeletal muscle microRNA expression with aging is associated with attenuated muscle plasticity and inhibition of IGF-1 signaling. FASEB J 28:4133–4147

Soriano-Arroquia, A., et al., *The functional consequences of age-related changes in microRNA expression in skeletal muscle.* Biogerontology, 2016. 17(3): p. 641-54.

**Key Resource Tables**

**Antibodies**

| **Reagent or resource** | **Source** | **Identifier** |
| --- | --- | --- |
| P62 | Cell Signaling | 3E11 |
| TOM20 (anti-mouse) | Abcam, UK | ab56783 |
| TOM20 (anti-rabbit) | Abcam, UK | ab186734 |
| LC3B | Abcam, UK | ab10912 |
| DJ-1 | Abcam, UK | ab76241 |
| PARKIN | Abcam, UK | ab15954 |
| PINK1 | Abcam, UK | ab23707 |
| anti-rabbit-Alexa488 | ThermoFisher Scientific, UK | A27034 |
| anti-mouse –Alexa488 | ThermoFisher Scientific, UK | A28175 |
| Anti-rabbit-Alexa532 | ThermoFisher Scientific, UK | A-11009 |
| Anti-mouse- Alexa532 | ThermoFisher Scientific, UK | A-11002 |
| WGA | Vector Laboratories | L-1020 |

**Chemicals**

| **Reagent or resource** | **Source** | **Identifier** |
| --- | --- | --- |
| DMEM | Sigma Aldrich, Dorset UK | [D5796](https://www.sigmaaldrich.com/catalog/product/sigma/d5796?lang=en&region=IE) |
| Fetal bovine serum | Sigma Aldrich, Dorset UK | F7524 |
| Horse serum | ThermoFisher Scientific, UK | 26050088 |
| CCCP | Sigma Aldrich, Dorset UK | C2759 |
| Bafilomcin A | Sigma, Ireland | B1793 |
| BSA | Sigma, Ireland | A2153 |
| Glycine | Sigma, Ireland | G8898 |
| Triton X | Sigma, Ireland | X100 (lab-grade) or T8787 (for mol biology) |
| DAPI, 1 μg/ml | Sigma Aldrich, Dorset UK | D9542 |
| Ammonium bicarbonate | Sigma Aldrich, Dorset UK | 40867 |
| Bradford assay | BioRad, UK | 500-0006 |
| 1% RapiGest | Waters, Manchester, UK | 186001861 |
| Tris(2-carboxyethyl)phosphine hydrochloride (TCEP) | Sigma Aldrich, Dorset UK | C4706 |
| N-ethylmaleimide | Sigma, Aldrich, Dorset UK | D4269 |
| Trypsin | Sigma, Ireland | T4049 |
| Trypsin Gold (Mass Spec) | Promega | V528A |
| 2-propanol | Sigma, Ireland | I9516 |
| Chloroform:isoamyl alcohol 24:1 | Sigma, Ireland | C0549 |
| Hydromount | National Diagnostics | HS-106 |
| Trizol | ThermoFisher Scientific, UK | 15596026 |

**Commercial kits**

| **Reagent or resource** | **Source** | **Identifier** |
| --- | --- | --- |
| SuperscriptII | ThermoFisher Scientific, UK | 18064014 |
| miRScript RT II | Qiagen, UK | 218161 |
| Sso Advanced SybrGreen | Biorad, UK | 1725271 |
| miRScript SybrGreen | Qiagen, UK | 218073 |
| Mitochondrial ToXGlo | Promega | [G8001](https://worldwide.promega.com/products/cell-health-assays/oxidative-stress-assays/mitochondrial-toxicity-assay/?catNum=G8001) |

**Experimental models**

| **Reagent or resource** | **Source** | **Identifier** |
| --- | --- | --- |
| Mice | Charles River | C57BL6/J |
| mitoQC | Dr. Viktor Korolchuk, Newcastle University | Allen *et al*, (2013) EMBO Reports. |

**Software**

| **Reagent or resource** | **Source** | **Identifier** |
| --- | --- | --- |
| ImageJ | Imagej.nih.gov | ImageJ |
| Olympus Fluoview3000 | Olympus; licence: NUI Galway | Olympus Fluoview3000 |
| Prism8 | Graphpad | Prism8 |
| Photoshop | Adobe | Photoshop CC release 2017.1.6 |
| AxioVision Software version 4.8 | Zeiss; licence: University of Liverpool | AxioVision Software version 4.8 |

**Table S2.** Sequences of primers used for qPCR.

| Gene name | Forward primer sequence | Reverse primer Sequence |
| --- | --- | --- |
| β2-microglobulin | GGAGAATGGGAAGCCGAACA | TCTCGATCCCAGTAGACGGT |
| Sirt-1 | GGCCGCGGATAGGTCCATA | CCACAGGAGACAGAAACCCC |
| Foxo-3 | AGTGGATGGTGCGCTGTGT | CTGTGCAGGGACAGGTTGT |
| P62 | GTGAATTCGCTCGCCGCTCGCTAT | CGTCTCGAGTGCCTGCTGACAACACCTA |
| Park2 | ACAGGGCTCCTGACATCTG | CAAGGACACGTCGGTAGCTT |
| DJ-1 | AACACACCCACTGGCTAAGG | GGGCTTGGGCTCTAGTCTTT |
| Pink1 | GCTTGCCAATCCCTTCTATG | CTCTCGCTGGAGCAGTGAC |
| Lc3b | AGCAGCATCCAACCAAAATC | CTGTGTCCGTTCACCAACAG |
| Opa1 | TCAGCAAAGCTTACATGCAGA | TGCTTGGACTGGCTACATTTT |
| Pgc-1α | TTCCACCAAGAGCAAGTAT | CGCTGTCCCATGAGGTATT |
| Nrf2 | TGGGCCCTGATGAGGGGCAGTG | TCCGCCAGCTACTCCAGGTTGG |
| Mfn1 | TGCCCTCTTGAGAGATGACC | AGAGCCGCTCATTCACCTTA |
| Mfn2 | GGGGCCTACATCCAAGAGAG | CCTTGGACAGGTACCCTTTG |
| Tfam | GCTGATGGGTATGGAGAAG | GAGCCGAATCATCCTTTGC |
| CoxIV | TGGGAGTGTTGTGAAGAGTGA | GCAGTGAAGCCGATGAAGAAC |
| Nd-1 | CCTATCACCCTTGCCATCAT | GAGGCTGTTGCTTGTGTGAC |
| Tom20 | AGTCGAGCGAAGATGGTGG | GCCTTTTGCGGTCGAAGTAG |
| CoxI | CACTAATAATCGGAGCCCCA | TTCATCCTGTTCCTGCTCCT |
| miR-181a | Cat. MS00006069 | Universal Primer, part of cat. 218073 |
| miR-181b | Cat. MS00006083 | Universal Primer, part of cat. 218073 |
| miR-181c | Cat. MS00001708 | Universal Primer, part of cat. 218073 |
| miR-181d | Ct. MS00011284 | Universal Primer, part of cat. 218073 |

**Table S3.** The sequences of murine p62 or Park2 3’UTRs or DJ-1 Exon 6 (wild type – WT or with mutations introduced into themiR-181a binding site – mutant) cloned into the GFP reporter constructs.

| Construct name | DNA Sequence |
| --- | --- |
| P62 WT | CCAAGCCCCACCCCCTTTGTCTTGTAGTTGCATCACGTAGAGCAGCAGGGCTTCTATAGATAGGCCCAGTGTCTTGGCATTCTTGTAGAATCTTCAGGTGGGAATGTGTGATGCCTTTTCAGGCAATAGGAAAGTGCATGAGGAGAGTTTTGAATGTGCATATGCTGACGCCTGAGAACAGACCCAGGTACCCGTGGCTGAGCTGAGCTTCCTCTGCTTTCCCTAGGCCTGGCCTCTGCAGGGAACTGCAGCACACACTGCACTCCCACCTGCTCTTGCCGCCAGCATTGCACCAGCAGTCCAGAATTCCTGCCTGACAACCCGTGTTTCCTTTATTAAAAGTGATTAGTACAACTGCTAGTTATTTTCAACAAATAAAGCCATTATGTTAAGAGGGGACTGTCCATAGTGAGTGAAAGGTGGCAGGCAGGGGCCTACAGCTCCTAGGGAATGGAGAATTCATGTGAAGCCGAATGAAGG |
| P62 Mutant | CCAAGCCCCACCCCCTTTGTCTTGTAGTTGCATCACGTAGAGCAGCAGGGCTTCTATAGATAGGCCCAGTGTCTTGGCATTCTTGTAGAATCTTCAGGTGGGAATGTGTGATGCCTTTTCAGGCAATAGGAAAGTGCATGAGGAGAGTTTTGCCTGTGCATATGCTGACGCCTGAGAACAGACCCAGGTACCCGTGGCTGAGCTGAGCTTCCTCTGCTTTCCCTAGGCCTGGCCTCTGCAGGGAACTGCAGCACACACTGCACTCCCACCTGCTCTTGCCGCCAGCATTGCACCAGCAGTCCAGAATTCCTGCCTGACAACCCGTGTTTCCTTTATTAAAAGTGATTAGTACAACTGCTAGTTATTTTCAACAAATAAAGCCATTATGTTAAGAGGGGACTGTCCATAGTGAGTGAAAGGTGGCAGGCAGGGGCCTACAGCTCCTAGGGAATGGAGAATTCATGTGAAGCCGAATGAAGG |
| Park2 WT | AGCCATTTCTTCTTCTCGATGCATATAAGCACATAAATGCGCACACACAAACACAGGCTGCAGATTACAGAAGCAGCCCCTAGATCCTTTCCAGGGCACCCACAGAAAACCACAGCACCCGCTGGCCCCAGGGGGAGGAGGCACTTTCAGCCTCTGGCTCACTCGAATGTCAGAGCTTAGATGAGGGTGCACCTTTGGTTTGGATTCTGTAGAAGCCATGAGTGAGGTGGGAAGTGTTTTCCAGGGTTGTTGCCACGCCCTGGGTAAGTAACACCTCTGAGGATTCTCAGAAGCACACTTGAGATCTGAGGAACGCTGCTCTCATGTAGTAATCATCTATTCCCAAAGGGCCCCCTGCAG |
| Park2 Mutant | AGCCATTTCTTCTTCTCGATGCATATAAGCACATAAATGCGCACACACAAACACAGGCTGCAGATTACAGAAGCAGCCCCTAGATCCTTTCCAGGGCACCCACAGAAAACCACAGCACCCGCTGGCCCCAGGGGGAGGAGGCACTTTCAGCCTCTGGCTCACTCGAGCAGTCAGAGCTTAGATGAGGGTGCACCTTTGGTTTGGATTCTGTAGAAGCCATGAGTGAGGTGGGAAGTGTTTTCCAGGGTTGTTGCCACGCCCTGGGTAAGTAACACCTCTGAGGATTCTCAGAAGCACACTTGAGATCTGAGGAACGCTGCTCTCATGTAGTAATCATCTATTCCCAAAGGGCCCCCTGCAG |
| DJ-1 WT | GTCCTACGGCTCTGTTGGCTCACGAAGTAGGTTTTGGATGCAAGGTCACAACACACCCACTGGCTAAGGACAAAATGATGAATGGCA |
| DJ-1 Mutant | GTCCTACGGCTCTGTTGGCTCACGAAGTAGGTTTTGGATGCAAGGTCACAACACACCCACTGGCTAAGGACAAAATGATGGGTGGCA |
